# Anatomical Investigation Reveals a Second Olfactory System in Locusts

**DOI:** 10.1002/cne.70197

**Published:** 2026-08-19

**Authors:** Zane N. Aldworth, Brian Kim, Kui Sun, Esther Ndungu, Vi Nguyen, Mark A. Stopfer

**Affiliations:** ^1^ Laboratory of Cellular and Synaptic Physiology NIH‐NICHD Bethesda Maryland USA; ^2^ Brown University–National Institutes of Health Neuroscience Graduate Partnership Program Providence Rhode Island USA

## Abstract

Olfaction in locusts offers a useful model for understanding how the brain encodes environmental information. While previous studies of olfaction in locusts have focused mainly on the antennal pathway, most insects, including locusts, possess olfactory receptors on their palps. Although they are traditionally considered to be gustatory or mechanosensory structures, accumulating molecular, electrophysiological, and behavioral evidence shows locust palps also mediate olfactory processing. The palps therefore initiate a second olfactory pathway, whose neural architecture and sensory functions remain largely unexplored. Here, we used anatomical approaches to characterize the chemosensory system of the palps in *Schistocerca americana*, mapping pathways from the peripheral sensilla through the brain to third‐order neurons. We found that sensory input from the palps projects to two distinct regions: the gnathal ganglion, which integrates gustatory input from multiple head appendages, and the glomerular lobe of the cerebral ganglion, which, traditionally thought of as a gustatory center, appears instead to receive only olfactory input. The glomerular lobe, in turn, provides olfactory input to the accessory calyx of the mushroom body, a region traditionally considered gustatory. Notably, the palp and antennal olfactory pathways remain anatomically segregated through at least the third‐order neurons. Our systematic characterization of the palp olfactory system anatomy challenges existing views of olfactory and gustatory integration in hemimetabolous insects and establishes a framework for comparative studies of antennal and palp‐based olfactory coding.

## Graphical abstract

We traced chemosensory pathways from locust palps through the central nervous system. Olfactory input from basiconic sensilla targets the glomerular lobe and accessory calyx, whereas gustatory input is restricted to gnathal circuits. Thus, the palp and antennal olfactory pathways form parallel but separate systems.

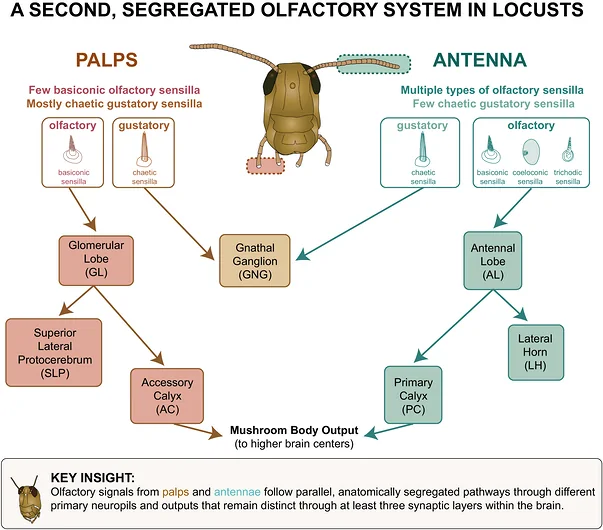

**Abbreviations**
ACAB: Accessory calyx bulb of the mushroom body
ACAC: Accessory calyx core of the mushroom body
ACAR: Accessory calyx ring of the mushroom body
ACKC: Accessory calyx Kenyon cell
AL: Antennal lobe
ALLN: Antennal lobe local neuron
ALPN: Antennal lobe projection neuron
AMMC: Antennal mechanosensory and motor center
AN: Antennal nerve
CA: Primary calyx
CB: Central body
CEC: Circumesophageal connective
CNS: Central nervous system
CRE: Crepine
CRG: Cerebral ganglion
CV: Cervical connective
DUM: Dorsal unpaired median neuron
GABA: Gamma‐aminobutyric acid
GCT: Glomerular lobe‐calycal tract
GL: Glomerular lobe
GLPN: Glomerular lobe projection neuron
GNG: Gnathal ganglion
KC: Kenyon cell
LH: Lateral horn
LS: Labial sensory center
LabMVT: Labial median ventral tract
LbN: Labial nerve
LbrN: Labral nerve
mALT: Medial antennal lobe tract
MC: Median crescent
ML: Medial lobe of the mushroom body
MS: Maxillary sensory center
MaxMVT: Maxillary median ventral tract
MnN: Mandibular nerve
MnS: Mandibular sensory center
MVTs: Median ventral tracts
MxN: Maxillary nerve
OBP: Odorant binding protein
OL: Optic lobe
OR: Olfactory receptor
OSN: Olfactory sensory neuron
PBS: Phosphate‐buffered saline
PED: Pedunculus
PN: Projection neuron
rALad: Cell body rind anterodorsal to the antennal lobe
rALld: Cell body rind laterodorsal to the antennal lobe
SEM: Scanning electron microscopy
SIP: Superior intermediate protocerebrum
SLP: Superior lateral protocerebrum
SMP: Superior medial protocerebrum
SPU: Spur of the mushroom body
TC: Tritocerebrum
VL: Ventral lobe of the mushroom body
VLP: Ventrolateral protocerebrum

## 1 Introduction

Chemosensation in locusts is a useful model for studying how the brain represents environmental information. The relative simplicity and accessibility of the locust brain have made it a valuable model for studying phenomena also observed in the sensory systems of the mammalian central nervous system—including coordinated, synchronous neural oscillations (Laurent and Davidowitz 1994; Laurent and Naraghi 1994; MacLeod and Laurent 1996; Stopfer and Laurent 1999), ensemble temporal coding (Laurent and Davidowitz 1994; Wehr and Laurent 1996; Mazor and Laurent 2005), and sparse neural responses (Perez‐Orive et al. 2002; Papadopoulou et al. 2011)—all of which are believed to help encode information. Much of this research has focused on the structures and functions of neural pathways beginning in the antenna and extending to central brain structures like the mushroom bodies and lateral horn (LH). However, like most insects, locusts possess a second set of chemosensory organs on their heads: the palps. These consist of two pairs of finger‐like appendages near the mouthparts that are thought to act as a single functional unit and play an essential role in food selection (Blaney and Chapman 1970). The neural pathways arising from the palps, their sensory sensitivities, and the mechanisms by which they encode information remain largely unexplored. The presence of two distinct chemosensory pathways, each originating from a different organ, offers a unique opportunity to examine and compare their structural and functional characteristics and their sensory coding strategies.

Although the olfactory role of the palps has been well established in holometabolous insects such as *Drosophila* (Singh and Nayak 1985; de Bruyne et al. 1999), palps in hemimetabolous insects like locusts have been viewed as mainly gustatory and mechanosensory (Frings and Frings 1949; Jawlowski 1954; Blaney and Chapman 1970; Ernst et al. 1977; Weiss 1981; Ignell et al. 2000; Homberg et al. 2004; Frambach and Schurmann 2004; Farris 2008). Electrophysiological recordings from sensilla on the tips of the palps, for example, show they respond to tastants (Haskell and Schoonhoven 1969; Blaney 1974, 1975; Blaney and Duckett 1975; Winstanley and Blaney 1978). However, early structural studies identified a population of sensilla on the palps’ distal tips that appeared to be olfactory (Blaney 1974, 1977; Jin et al. 2006), and more recent molecular studies have revealed the expression of olfactory receptors (ORs), olfactory binding proteins (OBPs), and other olfaction‐related molecules in the palps (Jin et al. 2006; Xu et al. 2013; Z. Wang et al. 2015; Zhang et al. 2017; H. Li, Wang, et al. 2018; Pregitzer et al. 2019; Lemke et al. 2020; Boronat‐Garcia et al. 2022). Further, electrophysiological studies have shown that olfactory sensory neurons (OSNs) on the palps respond to puffs of volatile chemicals (Zhang et al. 2017; H. Li, Wang, et al. 2018; H. Li, You, et al. 2018; L. Sun et al. 2022), and behavioral studies have shown that palp‐mediated detection of odorants can drive behavior (Zhang et al. 2017; P. Wang et al. 2019; L. Sun et al. 2022; Pan et al. 2023). However, beyond the sensory periphery, little is known about the neural architecture underlying chemosensation in locust palps. It remains unclear whether gustatory information from the palps is integrated with input from olfactory sensilla or whether the two sensory modalities are processed independently.

We used anatomical techniques to characterize the palp chemosensory system in the locust *Schistocerca americana*, starting with sensilla and extending to third‐order neurons. We also compared neurons associated with the palps and the antennae at analogous points along the two sensory pathways. We found that chemosensory information from the palps projects to two regions within the locust central nervous system: the gnathal ganglion (GNG), which contains the maxillary (MS) and labial (LS) sensory centers, and the glomerular lobe (GL) within the cerebral ganglion. We further found that the GL, a spheroidal neuropil composed of microglomeruli, integrates olfactory information from the palps, while the GNG integrates gustatory information from multiple sensory organs on the head, including the palps, antenna, labrum, and mandibles. Our findings challenge prevailing views of chemosensory processing in the locust by demonstrating that gustatory and olfactory information from the palps is processed in distinct brain regions, with only olfactory input entering the GL. Finally, we found that the olfactory pathway associated with the palps is completely separate from that originating in the antenna, at least through their respective third‐order neurons.

We concluded that the palps give rise to distinct and well‐separated gustatory and olfactory systems. Our point‐by‐point characterization of the first three layers in the palp olfactory pathway challenges existing models and lays the groundwork for comparative studies of sensory processing in the palp and antennal olfactory systems.

## 2 Materials and Methods

### 2.1 Standard Brain and Segmentation

To compare neurons and structures from different animals in a common coordinate system, we followed the methods of Heinze and colleagues to create a standard brain for *S. americana* (el Jundi and Heinze 2020).

Locusts of both sexes were selected from our crowded colony within 24 h of imaginal molt. Samples were obtained by decapitating the locust, removing the frons, clypeus, and labrum, and then perfusing the head capsule with insect saline. Fat and muscle tissue were removed, exposing the brain and GNG. Following dissection, the head ganglia were excised, fixed in 4% paraformaldehyde (PFA) overnight at 4°C, and then incubated in phalloidin (Abcam ab176753, 1:1000 dilution, *n* = 28 brains) for 2–5 days to stain neuropil. Tissues were then dehydrated in an ethanol series (30%, 50%, 70%, 80%, 90%, and 2 × 100% EtOH, 10 min each), cleared and mounted in methyl salicylate, and imaged on a Zeiss 710 confocal microscope with a 25x lens (Zeiss, 420852–9871). Image resolution was 0.66 µm in *x* and *y* and 3.0 µm in *z*. We tried two approaches to generating a standard brain: first, by creating a single average template using the Computational Morphometry ToolKit (CMTK, https://www.nitrc.org/projects/cmtk), and later, by using only the highest‐quality individual tissue sample from our dataset. The latter approach yielded better results. After selecting a standard brain, we used FIJI's segmentation editor plugin (https://imagej.net/plugins/segmentation‐editor) to delineate 20 different bilaterally paired brain regions as well as three bilaterally paired gnathal regions related to chemosensation. For reference, we used the atlases of the related species *Schistocerca gregaria* and *Locusta migratoria* and recommendations of the Insect Brain Name Working group as guides (Tyrer et al. 1982; Ito et al. 2014; von Hadeln et al. 2018). Regions delineated in the cerebral ganglion were: the mushroom body accessory calyx bulb (ACAB), accessory calyx ring (ACAR), accessory calyx core (ACAC), pedunculus of the mushroom body (PED), the primary calyx of the mushroom body (CA), the mushroom body spur (SPU), the ventral lobe of the mushroom body (VL, formally referred to as the alpha lobe), the medial lobe of the mushroom body (ML, formally referred to as the beta lobe), the central body (CB), the ventrolateral protocerebrum (VLP), the LH, the superior lateral protocerebrum (SLP), the superior intermediate protocerebrum (SIP), the superior medial protocerebrum (SMP), the crepine (CRE), the tritocerebrum (TC), the antennal mechanosensory and motor center (AMMC, combining the dorsal, lateral, and medial subregions of the AMMC as well as the ventral area of flagellar afferents), the median crescent (MC), the GL (previously referred to as the lobus glomerulatus), and the antennal lobe (AL). Regions defined in the GNG included the mandibular sensory center (MnS), the MS, and the LS, as well as the maxillary and labial median ventral tracts (MVTs). Volumes of segmented regions from the standard brain were measured using the 3D Objects Counter in FIJI. These volumes were compared with the same brain regions in seven other brains to confirm they were representative. The segmented neuropil regions are shown in Figure 2.

### 2.2 Scanning Electron Microscopy

To characterize sensilla on palps and antennae, we prepared SEM images from 13 female labial, 17 female maxillary, 11 male labial, and 11 male maxillary palps, all from the animals’ left side, and from a single male left and a single female right antenna. Samples were fixed with (2.5% glutaraldehyde and 1% formaldehyde in 0.12 M sodium cacodylate buffer, pH 7.2) for 24 h at room temperature (RT). The samples were then washed three times for 20 min with 0.1 M sodium cacodylate buffer, postfixed with 1% osmium tetroxide in 0.1 M sodium cacodylate buffer at RT, washed five times for 15 min with water, and then dehydrated in an ethanol series ending in 100% ethanol. The samples were critical point dried with CO_2_ in a Samdri‐795 critical point dryer (Tousimis Research Corp, Rockville, MD). Dried samples were mounted on SEM stubs with double‐sided carbon tape and then coated with 5 nm of gold in an EMS 575‐X sputter coater (Electron Microscopy Sciences, Hatfield, PA). Images were acquired with a Zeiss Crossbeam 540 microscope using the Secondary Electrons Secondary Ions (SESI) detector with a voltage of 1–3 kV and a current of 800 pA. Images were processed by Zeiss Smart SEM software (Carl Zeiss Microscopy GmbH, Jena, Germany). Palp tissues that showed signs of damage were not included.

### 2.3 Mass Dye Fills Into Palps, GL, AL, and MB

To identify brain regions innervated by palp afferents and their downstream synaptic partners, we used mass injection of dextran dyes with several different colored fluorophores (Molecular Probes D3308, D7162, D22912, and D22914), dextran conjugated to biotin (D7135), or neurobiotin (Vector SP‐1120). By using spectrally well‐separated fluorophores, we were able to unambiguously assign dye fill patterns to the respective injection sites, even when multiple dyes were used in the same tissue.

To deliver dye to the antennal, maxillary, and labial nerves, locusts were restrained, the tips of the maxillary or labial palp were removed, and a few dye crystals were inserted into the opening, which was then resealed with dental wax. To allow dye to perfuse into the tissues, locusts were then placed in a humidified chamber at RT overnight and then at 4°C for another 40 h. We filled five antennal nerves, 73 maxillary nerves, and 62 labial nerves from 50 different animals. Injection configurations included unilateral injections into the antennal nerve and maxillary palp, bilateral injections into the antennal nerves, unilateral injections into the maxillary or labial palp, unilateral injections into both the labial and maxillary palps, bilateral injections into both maxillary or both labial palps, or combined bilateral injections into both maxillary and labial palps. To distinguish projection patterns, different fluorophores were always used for each site during bilateral injections. In no case did we observe dyes staining neuropil contralateral to the injection site.

To apply dye to the mandibular or labral nerves, the head capsule was partially dissected to expose the relevant nerve. The nerve was then cut with sharp scissors, and the proximal end was drawn into a suction pipette filled with 2%–5% concentration (weight/volume) of dye mixed in insect saline. The locust was then placed in a humidified chamber at RT overnight prior to further dissection. We filled two labral nerves and two mandibular nerves in four animals. Injection configurations were always unilateral, with the mandibular/labral nerve injected with one color dye, and either the maxillary or labral nerve injected with a different color dye.

To apply dye to the GL, AL, and mushroom body, animals were decapitated, the frons was removed, and fat and muscle tissue were cleared away to expose the brain. Glass pipettes (dia. 15–30 µm) containing 2%–5% dye concentrations were inserted into the brain, and dye was ejected by puffs of air. Dye injection was performed in the following configurations: unilateral GL (*n* = 25); bilateral GL (*n* = 38); unilateral GL and accessory calyx (*n* = 2, different fluorophore used for each neuropil); and ipsilateral GL and AL with contralateral accessory calyx and primary calyx (*n* = 10, GL/AC using one fluorophore, AL/PC using different fluorophore). Following dye application, the head capsule was perfused with insect saline at RT for 1–2 h prior to subsequent dissection.

After dye delivery and perfusion, cerebral and gnathal ganglia were removed and then fixed in 4% PFA for 2 h at RT or overnight at 4°C. Tissues were then incubated with phalloidin 488 (Abcam ab176753, 1:1000 dilution) at 4°C for 40 h. Tissues containing neurobiotin or dextran biotin dye were incubated with Alexa Fluor 633‐conjugated streptavidin (ThermoFisher S21375). Following incubation, tissues were dehydrated in an ethanol series, cleared in methyl salicylate, and imaged in whole mount with a confocal microscope (Zeiss models 710, 800, 880, or 900 in the NICHD Microscopy and Imaging Core). For some samples, tissues were then re‐hydrated in ethanol, dehydrated in an ascending sucrose series (overnight each in 10%, 20%, and 30% sucrose), embedded in OCT compound (Tissue‐Tek, 27050), frozen in 2‐methylbutane on dry ice, and then transversely sectioned (30 µm) on a Leica model CM3050 S cryostat. Higher‐resolution images of axons in tracts were obtained with the Airyscan function on a Zeiss 880 confocal microscope (Wu and Hammer 2021).

### 2.4 Immunohistochemistry

To immunolabel for GABA, whole brains were removed from the head capsule and washed twice for 20 min each in PBS solution at RT, blocked overnight at 4°C in 5% goat serum in PBS with 1% triton, and then incubated in primary antibody: either mouse ⍺‐GABA (Sigma A0310, RRID:AB_476667) or rabbit ⍺‐GABA (Sigma A2052, RRID:AB_477652) at 1:100 dilutions. Primary incubation lasted 7 days at 4°C, followed by two 20‐min washes in PBS. Secondary antibodies with fluorescent fluorophores were used at 1:100 dilutions (Invitrogen A‐1108, RRID:AB_143165; Sigma SAB4600312, RRID:AB_2936984), mixed with phalloidin and DAPI (Invitrogen, D3571) to visualize neuropils and neuron somata, respectively. Secondary incubation lasted 7 days at 4°C, after which brains were dehydrated, cleared, mounted in wholemount, and imaged.

### 2.5 Single Neuron Dye Fills

To fill projection neurons with dye, we used patch pipettes (*R* = 5–10 MΩ) to obtain high‐resistance (1 GΩ) seals from the somata of AL neurons in either anterior or posterior clusters. To access neurons with somata in the cell body rind laterodorsal to the antennal lobe (rALld cluster), the AL was gently removed following protease application. Intracellular solutions loaded into the recording pipette (Laurent et al. 1993) contained either 0.5% (w/v) neurobiotin (Vector SP‐1120), Lucifer Yellow (Molecular Probes L453), or biocytin 546 (Fisher a12923). In our hands, only neurobiotin reliably filled axons. Dye was injected by passing hyperpolarizing current steps (10–20 pA, 1 Hz, 50% duty cycle) for 5–30 min. Brains were then removed and fixed overnight in 4% PFA at 4°C. After filling neurons with neurobiotin, the brains were incubated with streptavidin‐conjugated Alexa Fluor and Phalloidin for 2–5 days. Following fixation and incubation, brains were dehydrated, cleared, mounted, and imaged as for mass dye fills.

To make single sensillum dye fills, we used an approach modified from that used for olfactory sensilla recordings on the antenna and palps of locusts (Hansson et al. 1996; Raman et al. 2010; Zhang et al. 2017; H. Li, You, et al. 2018; L. Sun et al. 2022; Kim et al. 2023). Quartz electrodes (10–20 MΩ) filled with an electrode solution containing 2%–5% (w/v) neurobiotin in locust saline were gently inserted at the base of the sensillum, and a chlorided silver grounding wire was inserted into the contralateral eye. Dye was loaded into the sensillum via current injection (0.5 nA, 1 Hz, 50% duty cycle) for 30–90 min. Locusts were then placed in humidified chambers at 4°C for 3–5 days before brains were removed and fixed overnight in PFA. Conjugation of neurobiotin, clearing, mounting, and imaging followed the same procedure as for mass dye fills. We were unable to successfully fill chaetic sensilla using this method. However, to check whether neurons from chaetic sensilla ascended through the MVTs to the brain, we filled the circumesophageal connective in three animals with a 2%–5% neurobiotin solution using a suction pipette. Dye was allowed to load overnight while the animals were stored in humidified chambers at RT, and then over a second night at 4°C, after which the GNG and ipsilateral palps were removed, fixed, conjugated with streptavidin, dehydrated, cleared, and mounted as described for mass dye fills. Labeled sensilla on the ipsilateral palp tip were then carefully examined to determine whether they were chaetic or basiconic.

### 2.6 Computation and Statistical Analysis

Sensilla were counted in SEM images of the palps and antenna by the cell counter plugin to FIJI (https://imagej.net/plugins/cell‐counter). Soma from dextran fills were counted from confocal stacks using the multi‐point tool in FIJI (https://imagej.net/ij/docs/guide/146‐19.html#toc‐Subsection‐19.6). To register neural tissue into the coordinate system of the standard brain, CMTK was used to align the Phalloidin channel of confocal stacks. Tracing and reconstructing single‐neuron fills were done with the Simple Neurite Tracer plugin in FIJI (https://imagej.net/plugins/snt/) or Neutube (https://neutracing.com/). ANOVA tests comparing sensilla counts with tissue types were performed in Matlab using the “anovan” function. Wilcoxon rank sum tests comparing the diameters of the MVTs for maxillary versus labial axons were performed in Matlab using the “ranksum” function.

Numbers of axons in the MVTs were estimated using both an upper bound and a lower bound method. For the upper bound estimate, we divided the number of axons from neurons in the tip of the palp, 2300 (Blaney and Chapman 1969b; Blaney et al. 1971; Jin et al. 2006), by the cross‐sectional area of the maxillary nerve in the tip of the palp, 1000 µm^2^ (Blaney and Chapman 1969a), and multiplied the result by the measured cross‐sectional area of the MVT, 145.4 µm^2^. For the lower bound estimate, we counted the axon profiles in our most complete dye fill of the MVT using the “analyze particles” function in FIJI (https://imagej.net/imaging/particle‐analysis).

To estimate the number of microglomeruli in the AL and GL, microglomeruli were first manually segmented in a single medial slice of each lobe. We later verified that microglomerular density was approximately uniform across neighboring sections. To extrapolate from the number of microglomeruli profiles in a single slice to the number contained within the full lobe volume, we used:

$$N_{\mathrm{mg}}=\frac{\mathrm{Vo}l_{\mathrm{lobe}}}{{\bar{\mathrm{Vol}}}_{\mathrm{mg}}}\times \frac{\sum A_{\mathrm{mg}}}{A_{\mathrm{lobe}}},$$

where *A*_lobe_ and *A*_mg_ represent the measured area of the AL or GL and the individual microglomeruli within the slice, respectively, and Vol_lobe_ is the full volume of the AL or GL measured from the 3D reconstruction. The term Vol_mg_ represents the volume of a microglomerulus, approximated as a sphere from the mean cross‐sectional area as Vol_mg_ = 4/3 × *π* × (*A*_mg_/*π*)^3/2^. Because a single optical section intersects microglomeruli at random positions relative to their maximal diameter, the measured cross‐sectional areas systematically underestimate the true maximal cross‐sectional area. Under assumptions of approximately isotropic microglomerular shapes, the expected mean observed cross‐sectional area is two‐thirds of the maximal cross‐sectional area. We therefore corrected the *A*_mg_ term by a factor of 3/2 prior to estimating microglomerular volume (Wicksell 1925). The term ∑*A*_mg_/*A*_lobe_ provides an estimate of the fractional area occupied by microglomeruli within the optical section, which was used as a proxy for the volumetric packing fraction within the lobe.

The total size of the glomerular lobe projection neuron (GLPN) population was estimated using two different methods. For Method 1, we counted the number of somata in the cluster dorsolateral to the AL stained by dextran injections into the GL. Somas were counted across full *z*‐stacks to avoid double counting of partially labeled cells. This was treated as a lower‐bound estimate, as not all somata take up dye during the injection process. For Method 2, we measured *r*_2_, the radius of GLPN axons from single neuron fills at the point where the glomerular lobe‐calycal tract (GCT) separates from the medial antennal lobe tract (mALT), and *r*_1_, the radius of the GCT in phalloidin‐labeled brains measured at the same point. We then used a hexagonal close‐packing approximation and adjustment for boundary effects (Lubachevsky and Graham 1997) to estimate how many circles of radius *r*_2_ could fit in a larger circle of radius *r*_1_ using the following equation:

$$n_{\mathrm{axons}}\approx \frac{\pi r_{1}^{2}}{2\sqrt{3}r_{2}^{2}}-\frac{\pi r_{1}}{2r_{2}}.$$

This assumes idealized circular cross‐sections and maximal packing density, and so was treated as an upper‐bound estimate, as actual packing density is expected to be lower due to irregular shape and spatial heterogeneity of axons.

## 3 Results

Our results reveal a second olfactory pathway in the locust, originating in basiconic sensilla on the maxillary and labial palps, projecting via the GL to the accessory calyx of the mushroom body. The following sections trace this pathway from the sensory periphery to higher brain centers and contrast its components with the well‐characterized antennal olfactory system.

### 3.1 Locust Palps Express Two Types of Chemosensory Sensilla Also Found on Antennae

**FIGURE 1:**
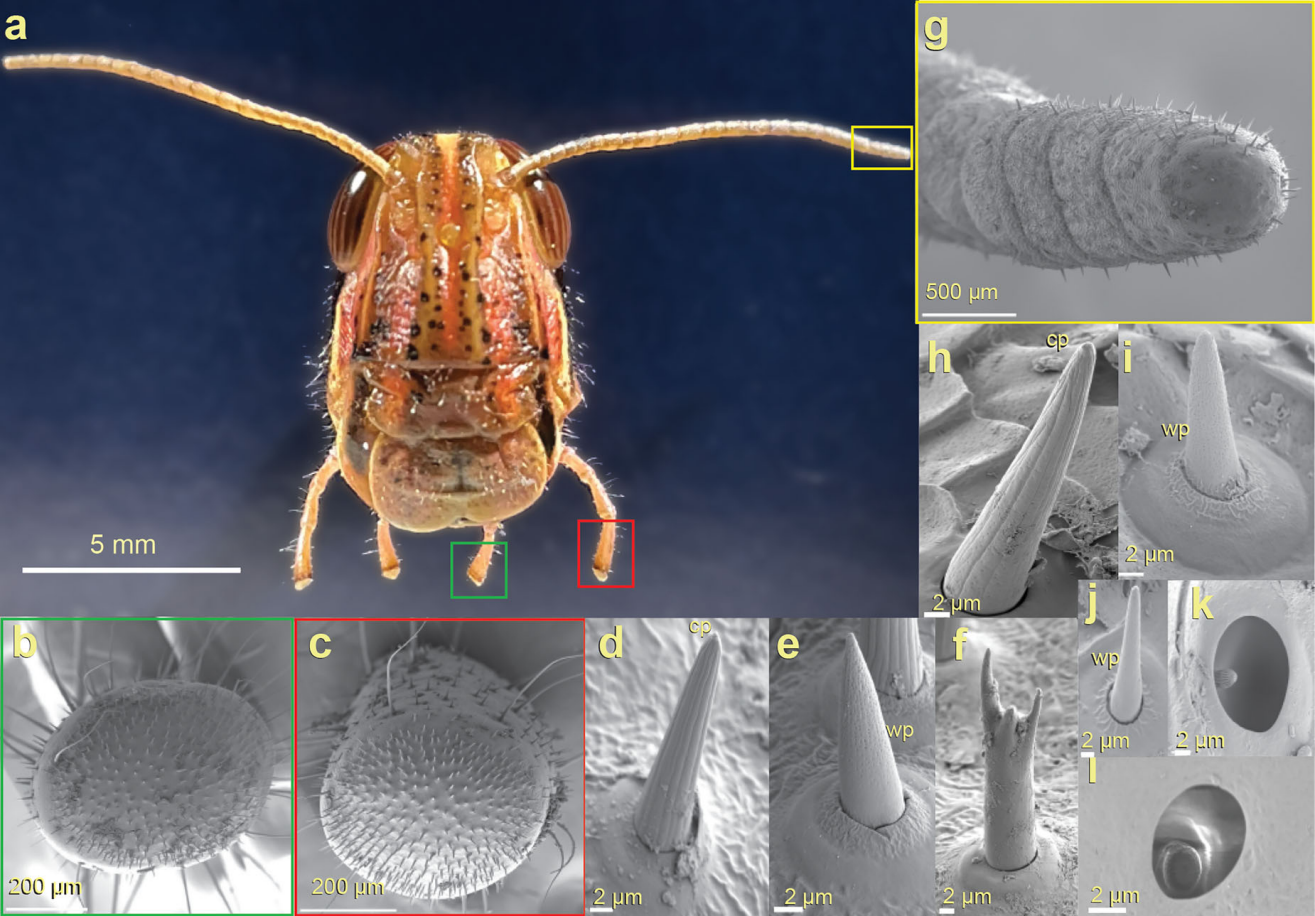
Chemosensilla cover the antennae and palps of *Schistocerca americana*. (a) Head of the locust *S. americana*, with boxes highlighting the antenna (yellow), labial (green), and maxillary (red) palps. (b–l) SEM images of chemosensory organs and sensilla. (b, c) SEM of the sensillar cap on the terminal end of the labial and maxillary palps, respectively. (d) SEM of the chaetic sensillum of the palp, a primarily gustatory sensillum with longitudinal ridges and a single apical crest pore (cp). (e) Basiconic sensillum of the palp, an olfactory sensillum covered with many wall pores (wp). (f) Central sensillum of the palp, a smooth sensillum lacking any ridges or pores, sometimes bearing multiple peaks, of unknown sensory modality. (g) SEM of antennal segments 1–6 (numbered distal to proximal). (h) Chaetic sensillum of the antenna, mainly gustatory, with longitudinal ridges and a single apical crest pore (cp). (i) Basiconic sensillum of the antenna, an olfactory sensillum covered with many wall pores (wp). (j) Trichodic sensillum of the antenna, an olfactory sensillum covered with many wall pores (wp). (k, l) Coeloconic sensilla of the antenna, located inside cuticular pits. Sensilla with longitudinal ridges (k) contain neurons sensitive to odors, while poreless sensilla (l) contain hygrosensitive neurons. Scale bars: 5 mm (a), 500 µm (g), 200 µm (b, c), and 2 µm (d–f, h–l).

We began our characterization of palp‐based chemosensory pathways by examining sensillar morphology and distribution, using the antennae as a comparative framework. Previous work has suggested that palp pathways combine gustatory and olfactory inputs, but the cellular origins of these pathways remain incompletely defined. The palps of locusts and related orthopterans end in dome‐like structures densely covered by sensillar hairs (Figure 1a–c). To characterize these sensory arrays, we made and analyzed scanning electron microscope (SEM) images of the sensilla on the terminal domes of each palp (Figure 1b,c) and, for comparison, the sensilla on antennae (Figure 1g).

The tips of the maxillary and labial palps are primarily covered by chaetic sensilla (Figure 1d), each featuring pronounced longitudinal grooves and a single apical crest pore. Each palp also has a small population of basiconic sensilla (Figure 1e), which are conical and covered with wall pores. We also found that each palp contains a single sensillum that had not been previously described. Always located near the center of the palp dome, this relatively large, smooth sensillum lacks visible pores, suggesting it serves a mechanosensory function (Figure 1f). These novel sensilla frequently forked into two or more prongs at their apical termini. The palps lack the trichoid, coeloconic, and club‐shaped sensilla that have been described as chemosensors on the antennae.

Prior electrophysiological studies have shown that neurons in palp chaetic sensilla respond to tastants, while neurons in palp basiconic sensilla respond to odors (Haskell and Schoonhoven 1969; Blaney 1974, 1975; Blaney and Duckett 1975; Zhang et al. 2017; H. Li, You, et al. 2018; L. Sun et al. 2022). No physiological studies to date have investigated the novel central sensillum.

We compared the chemosensory sensilla array of the palps with that of the antennae, which, as previously reported (Slifer et al. 1957; Slifer et al. 1959; Chapman 1982; Greenwood and Chapman 1984; Chapman and Lee 1991; Ochieng et al. 1998; Chapman 2002; Nakano et al. 2023), contained five types of chemosensory sensilla. The chaetic sensilla on the antennae are longer and more rod‐shaped than those on the palps and feature faint longitudinal grooves along their external cuticle (Figure 1h). The basiconic sensilla of the antennae (Figure 1i) are less tapered and possess an even higher surface density of wall pores than their counterparts on the palps. Trichoid sensilla (Figure 1j), which are not found on the palps, are short and slender with wall pores covering the surface and are sensitive to odors. Finally, the antennae surface contains coeloconic sensilla, which are short, located within cuticular pits, and can be subdivided into double‐walled sensilla with ridges (Figure 1k), which are sensitive to odors, and poreless, club‐shaped sensilla (Figure 1l), which are hygrosensitive (Altner et al. 1981).

In all, we examined 20 maxillary and labial palps from both sexes (Table 1). Each palp contained 338.5 ± 9.8 chaetic sensilla (mean ± SE), 10.9 ± 0.2 basiconic sensilla, and exactly one central sensillum (Table 1), regardless of sex. While there were no differences in the numbers of basiconic sensilla across sex or palp type, we did find significant differences in the number of chaetic sensilla: maxillary palps contained a few more sensilla than labial palps (354.5 ± 10.9 vs. 322.5 ± 15.2, for chaetic sensilla, Table 1; 11.3 ± 0.2 vs. 10.4 ± 0.3, for basiconic sensilla, Table 1), and palps from females had significantly more chaetic sensilla than those from males (364.2 ± 12.0 vs. 312.8 ± 10.8, Table 1), consistent with the larger size of female locusts and with a similar analysis of labial palps in *L. migratoria* (Jin et al. 2006).

**TABLE 1:** Mean sensilla counts for each tissue type.

Tissue type	*n*	Chaetic		Basiconic		Central	
		Mean	SE	Mean	SE	Mean	SE
Female labial	5	354.6	19.6	10.2	0.4	1.0	0.0
Female maxillary	5	373.8	14.7	11.4	0.2	1.0	0.0
Male labial	5	290.4	11.9	10.6	0.4	1.0	0.0
Male maxillary	5	335.2	11.5	11.2	0.4	1.0	0.0
All tissues	20	338.5	9.8	10.9	0.2	1.0	0.0

We used these sensilla counts to estimate the number of neurons projecting from the tip of the palps into the central nervous system. While mechanosensitive sensilla in insects are usually innervated by a single neuron, chemosensitive sensilla are usually innervated by several (Shields 2008). In locusts, each palp chaetic sensilla houses either five, six, or 10 gustatory sensory neurons and a single mechanosensory neuron (Blaney and Chapman 1969b; Blaney et al. 1971; Jin et al. 2006), while palp basiconic sensilla house around 15 sensory neurons, all olfactory (Blaney 1977; Jin et al. 2006). By multiplying our sensilla counts by the expected numbers of neurons per sensillum, we estimate information entering the locust central nervous system is carried in the axons of about 1800 gustatory sensory neurons, 165 OSNs, and 340 mechanosensory neurons, or roughly 2300 neurons in total, per palp, a number roughly consistent with prior estimates of 7270–8072 neurons in all four palps (two maxillary and two labial) in *L. migratoria* (Chapman and Thomas 1977; Chapman 1982).

### 3.2 Neurons in Chemosensory Sensilla on the Palps Innervate the Gnathal Ganglion and GL

**FIGURE 2:**
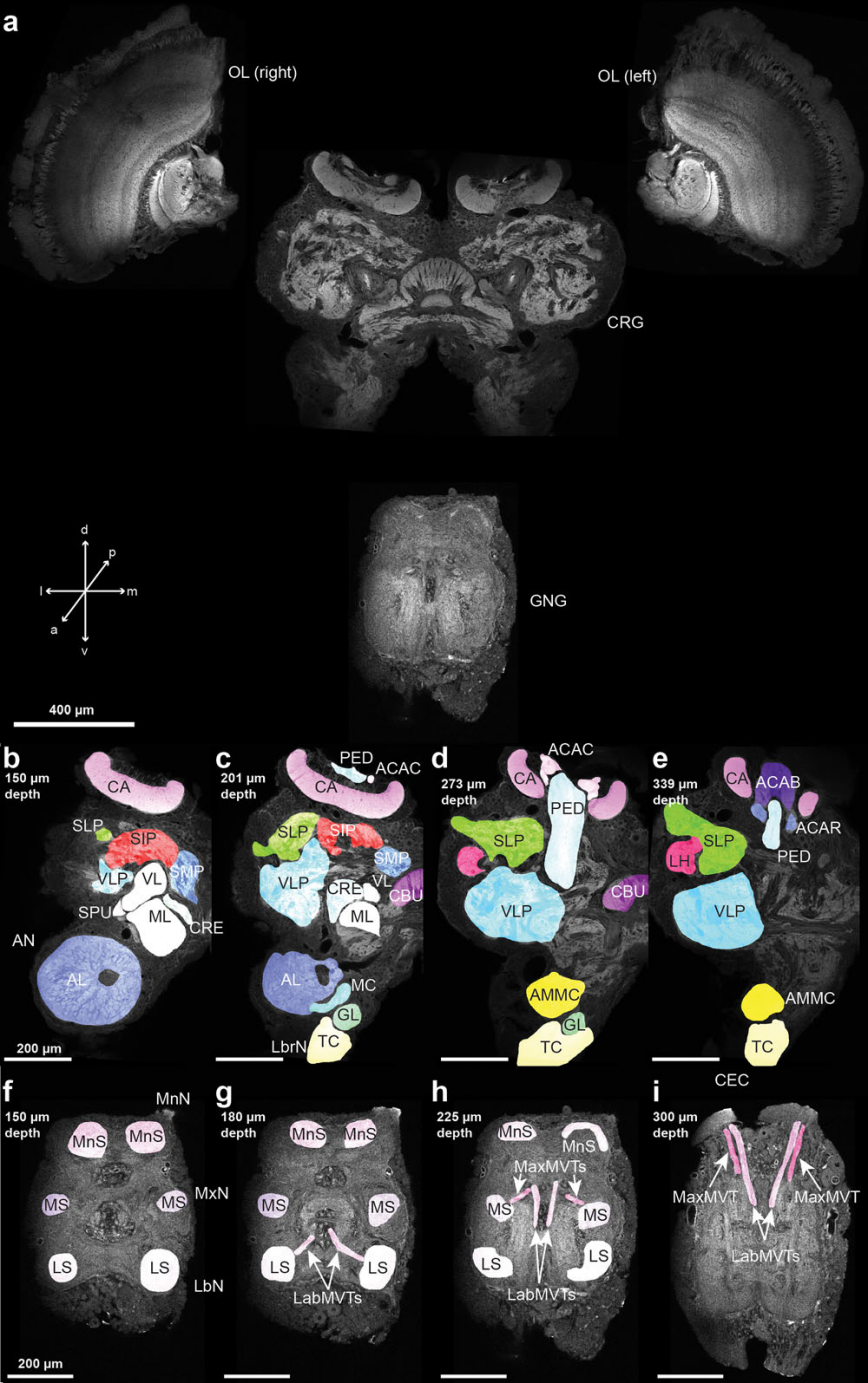
*Schistocerca americana* standard brain atlas. (a) Confocal image in the frontal section of the phalloidin‐stained tissue used to create the standard brain atlas of *S. americana*. The tissue was derived from separate animals for the optic lobes, cerebral ganglion, and gnathal ganglion. (b–e) Frontal sections of the cerebral ganglion at depths of 150, 201, 273, and 339 µm from the anterior surface of the tissue, respectively. Segmented neuropils for regions mentioned in this study are highlighted in color. (f–i) Frontal sections of the gnathal ganglion at depths of 150, 180, 225, and 300 µm from the anterior surface of the tissue, respectively. The segmented sensory centers and the respective median ventral tracts that carry axons to the cerebral ganglion via the cervical connective are highlighted in color. OL, optic lobe; CRG, cerebral ganglion; GNG, gnathal ganglion; CA, primary calyx; SLP, superior lateral protocerebrum; SIP, superior intermediate protocerebrum; SMP, superior medial protocerebrum; VLP, ventrolateral protocerebrum; SPU, spur of the mushroom body; VL, ventral lobe of the mushroom body; ML, medial lobe of the mushroom body; CRE, crepine; AL, antennal lobe; PED, pedunculus; ACAC, accessory calyx core of the mushroom body; CBU, central body, upper portion; MC, median crescent; GL, glomerular lobe; TC, tritocerebrum; AMMC, antennal mechanosensory and motor center; ACAB, accessory calyx bulb of the mushroom body; ACAR, accessory calyx ring of the mushroom body; LH, lateral horn; MnS, mandibular sensory center; MS, maxillary sensory center; LS, labial sensory center; AN, antennal nerve; LbrN, labral nerve; CEC, circumesophageal connective; MnN, mandibular nerve; MxN, maxillary nerve; LbN, labial nerve; MaxMVT, maxillary median ventral tract; LabMVT, labial median ventral tract. Scale bars: 400 µm (a) and 200 µm (b–i).

**FIGURE 3:**
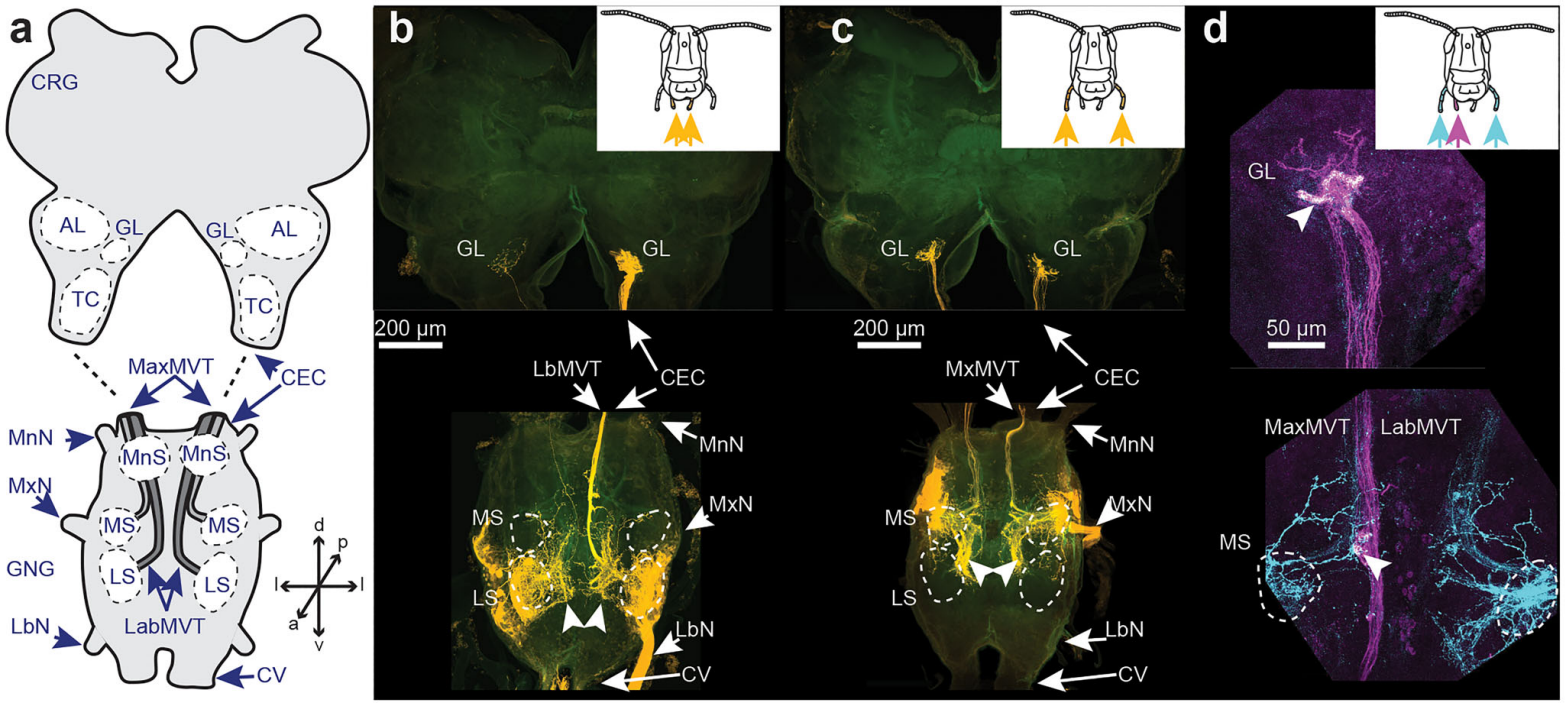
Dye injections reveal the palp olfactory pathway in the locust brain. (a) Schematic outlines of locust cerebral ganglion (CRG, upper) and gnathal ganglion (GNG, lower) in frontal views. Regions in dotted outlines represent relevant neuropils, including the antennal lobe (AL), glomerular lobe (GL), and tritocerebrum (TC) in the CRG, and the mandibular sensory center (MnS), maxillary sensory center (MS), and labial sensory center (LS) of the GNG. Also shown are the medioventral tracts connecting the labial (LabMVT) and maxillary (MaxMVT) sensory centers with the circumesophageal connective (CEC), which connects the GNG to the CRG. At the bottom, the cervical connective (CV) connects the two head ganglia to the thoracic ganglia. (b) Projection through a confocal stack of the CRG (upper) and GNG (lower) showing bilateral mass dye fills of the labial nerves originating in the labial palps. Neuropil regions are visualized by phalloidin tagging of actin (green), while the dye is visualized in orange. Arrowheads indicate prominent branching outside the LS. Inset shows a schematic of the locust head with both labial palps in orange. (c) Projection through a confocal stack of the CRG (upper) and GNG (lower) showing bilateral mass dye fills of the maxillary nerves originating in the maxillary palps from a different animal than that in (b). Arrowheads indicate prominent branching outside the MS. Inset shows a schematic of the locust head with both maxillary palps in orange. (d) Projection through a confocal stack of the area around the GL (upper) and the MS (lower) showing bilateral mass dye fills of the maxillary nerves (cyan) and a unilateral mass dye fill of the labial nerve (magenta), from a different animal than that in (b) or (c). Arrowheads indicate regions of overlap between arborizations of the two nerves (white). Scale bars: 200 µm (b, c) and 50 µm (d).

Palp chemosensory neurons project via dedicated nerves to the GNG, and a subset continues further to innervate the GL, a microglomerular neuropil distinct from the AL. Specifically, the left and right labial palps are each innervated by the labial nerves (LbN), and the left and right maxillary palps are each innervated by the maxillary nerves (MxN) (Blaney and Chapman 1969a). To facilitate descriptions of how palp nerves innervate the locust CNS, we constructed a standard brain for *S. americana* (Figure 2; see Section 2). The two pairs of palp nerves project directly to the GNG (Figure 3a), an ellipse‐shaped subesophageal structure connected dorsally to the cerebral ganglion (subsequently referred to as “the brain”) via the two thick circumesophageal connectives, and connected ventrally to the thoracic ganglia and ventral nerve cord via the cervical connectives (Tyrer et al. 1982). In addition to receiving the paired maxillary and labial nerves, the GNG is innervated by a pair of mandibular nerves (MnN) projecting from the mandibles. Each of the large nerves entering the GNG ipsilaterally innervates a corresponding region of sensory neuropil: the mandibular nerve leads to the MnS, the maxillary nerve leads to the MS, and the labial nerve leads to the LS.

To identify the central nervous system targets of palp output, we applied dextran‐conjugated dyes or neurobiotin to 88 maxillary and labial nerves in 41 animals, using phalloidin as a counterstain. When staining multiple nerves in the same tissue, we applied different‐colored fluorescent conjugates to each, allowing us to unambiguously link stained neuropils with the nerves innervating them (see Section 2). In 66 of the nerves, we found labeled sensory axons leading into the GNG. In many cases, nearly every phalloidin‐labeled axon in the nerve was also labeled by fluorescent dye as it entered the GNG, indicating our tracers were nonselectively taken up by the various axon types within the nerve (e.g., right side of Figure 3b). While most of the dendritic branching was contained within the MS and LS, we also often observed some projections from stained labial neurons running medially, dorsally, and anteriorly from the LS, and projections from stained maxillary nerves running medially, ventrally, and anteriorly from the MS (arrowheads in Figure 3b,c).

In 26 of the 66 fills, we observed labeled axons from both the maxillary and labial palps traveling ventrally to dorsally through the GNG, in fiber bundles called the medial ventral tracts (MVTs) (Tyrer et al. 1982) (Figure 3a). These axons exited the GNG through the circumesophageal connective and subsequently entered the brain. In all observed cases, there appeared to be more stained processes in the palp nerve entering the GNG than stained processes in the corresponding medial ventral tract. The tracts themselves remained segregated from one another, though some collaterals from labial medial ventral tracts overlapped with neural processes in the MS (Figure 3d).

Within the brain, the axons densely innervated a spherical neuropil known as the GL (Figure 3) just posterior, medial, and ventral to the AL. In preparations where the maxillary and labial nerves were labeled with different‐colored dyes, their axons sent processes to overlapping regions within the GL (Figure 3d). This region, formerly called the lobus glomerulatus (Jawlowski 1954; Satija 1958; Ernst et al. 1977; Ignell et al. 2000; Homberg et al. 2004; Frambach and Schurmann 2004; von Hadeln et al. 2018; L. Sun et al. 2022), has a microglomerular organization. In our standard brain (Figure 2), we measured the volume of the GL to be approximately 570,000 µm^3^ and found it to contain approximately 690 microglomeruli (see Section 2). In comparison, the AL volume from the same brain was approximately 8,900,000 µm^3^, and it contained approximately 810 microglomeruli, consistent with previous estimates from locusts (Ernst et al. 1977; Laurent and Naraghi 1994; Ignell et al. 2001; Anton et al. 2002; Ott and Rogers 2010).

### 3.3 The GL Is Not Innervated by Contact Chemosensory Neurons From Other Organs on the Head

**FIGURE 4:**
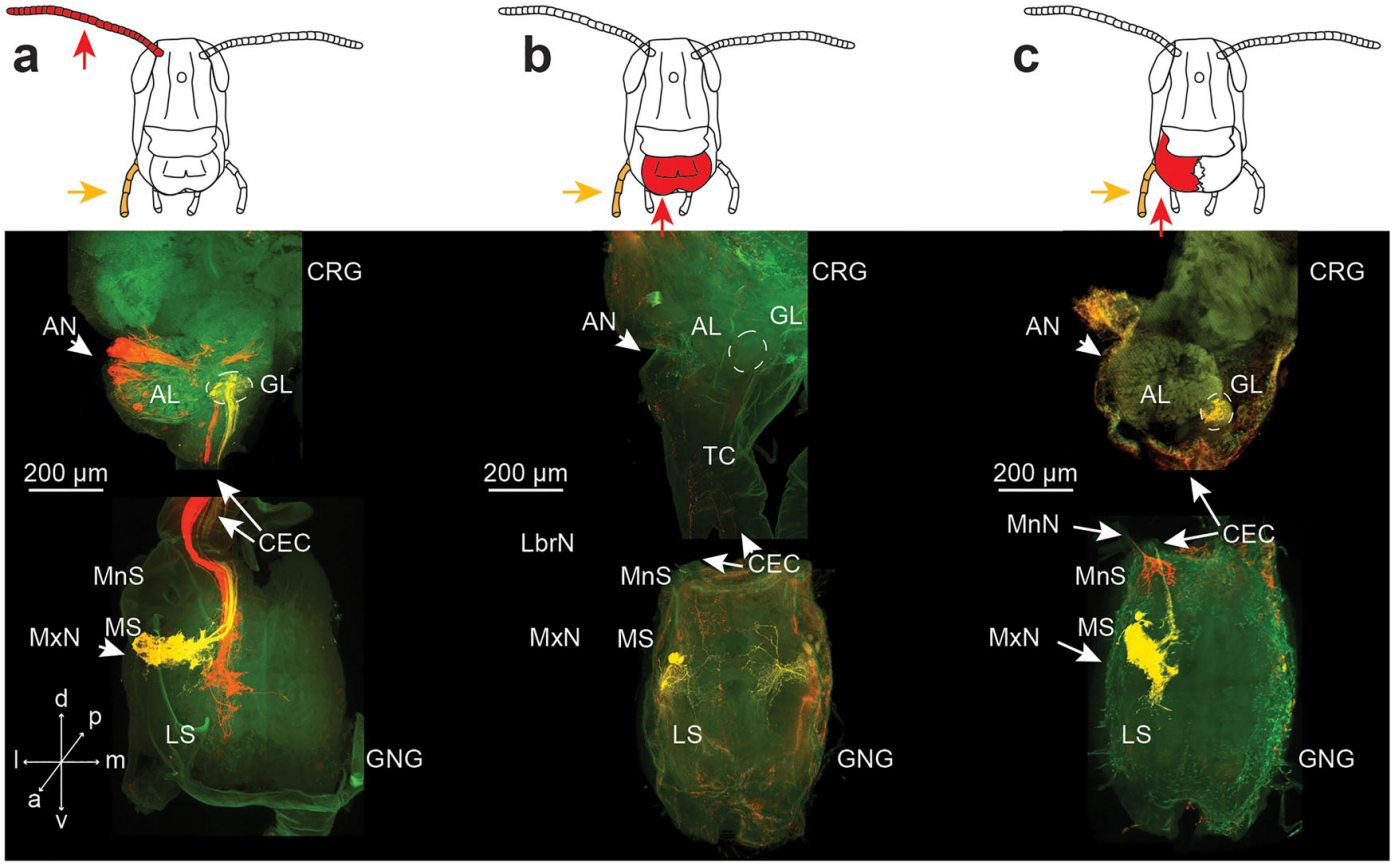
Among chemosensory organs on the head, only the palps send neuronal processes to the glomerular lobe. (a) Projection, frontal view, through a confocal stack of dye injections into the antennal nerves (AN, red) and maxillary nerve (MxN, orange), showing the innervated regions in the cerebral ganglion (CRG, middle) and gnathal ganglion (GNG, bottom). Top schematic shows the organs into which the dye was injected. (b) Dye injections into the labral nerve (LbrN, red) and maxillary nerve (orange); conventions as in (a). (c) Dye injections into the mandibular nerve (MnN, red) and maxillary nerve (orange); conventions as in (a). Neuropil regions are visualized by phalloidin tagging of actin (green). AL, antennal lobe; GL, glomerular lobe; CEC, circumesophageal connective; MnS, mandibular sensory center; MS, maxillary sensory center; LS, labial sensory center; TC, tritocerebrum. Scale bars: 200 µm (a–c).

To determine whether the GL serves as a broad chemosensory integration center, we examined whether it receives projections from other chemosensory structures: the antenna, labrum, and mandibles. To determine whether axons from antennal chaetic sensilla innervated the GL, we made mass stains of antennal nerves in three animals. To compare both antennal and palp neuron projection patterns in the same animals, we used a second marker to stain the maxillary nerves (Figure 4a). Stains from the antennal nerve strongly labeled the AL, and also a tract that exited it, bypassed the GL, and descended through the circumesophageal connectives to arborize in a region within the GNG medial to both the MS and LS (Figure 4a, lower panel), in agreement with labeling of single chaetic sensilla on the antennae of cockroaches (Nishino et al. 2005). Thus, neurons from contact chemosensory sensilla on the antennae do not project to the GL.

Gustatory sensilla have been identified on the labrum and mandibles, in addition to the antennae and palps (Thomas 1966; Haskell and Schoonhoven 1969; Louveaux 1975; Cook 1977; Bland 1982; Boyan et al. 2003; Zaim et al. 2013). To determine whether these sensilla project to the GL, we mass‐filled the labral nerve in four animals (Figure 4b) and the mandibular nerve in two animals (Figure 4c). The labral nerve projected mainly to the neuropil of the TC, ventral to the GL. But a few processes also descended through the circumesophageal connectives into the GNG, traveling to the ventral margins of the ganglion, extending collaterals at several points (Figure 4b). We observed no processes traveling dorsally from the TC to the GL.

Similarly, fills from the mandibular nerve densely stained the MnS, but did not appear to project into other regions of the GNG or via ascending tracts through the circumesophageal connectives toward the GL (Figure 4c).

These results show that, although the GL receives projections from chemosensory sensilla on the palp, it does not serve as a general integration center for chemosensory input. Neurons from contact chemosensory sensilla on the antenna, labrum, and mandibles all circumvent the GL and instead arborize within distinct regions of the TC and the GNG neuropil. Based on these findings, we propose that the widely branching neurons within the GNG enable this region to function as an integration center for gustatory information from multiple head organs (Braunig 1991), while the GL serves a more specialized role, processing chemosensory input exclusively from the palps.

### 3.4 A Minor Portion of the Projections to the Gnathal Ganglion Terminates in the GL

**FIGURE 5:**
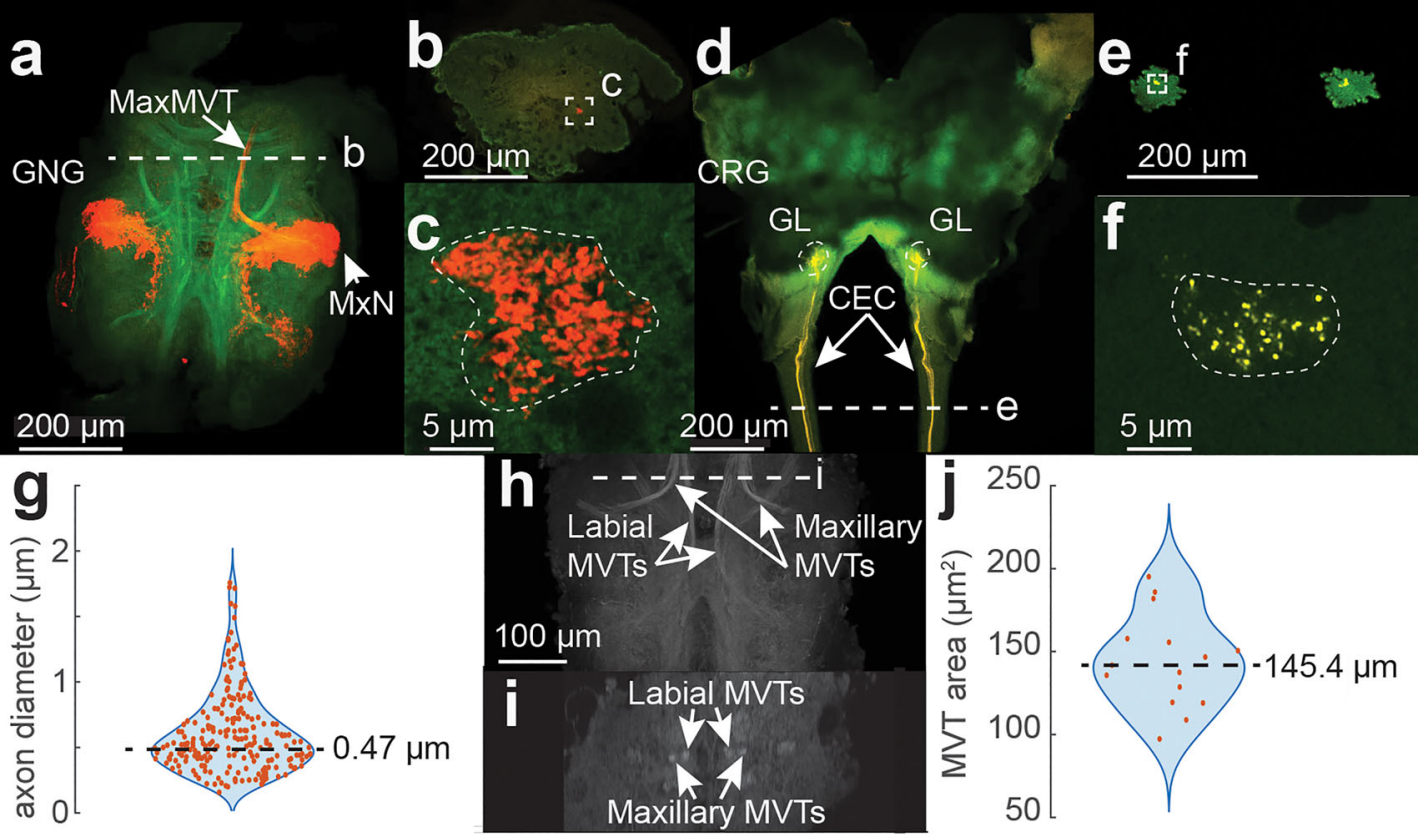
Ascending tracts innervated by palp neurons contain several dozen axons. (**a**) Projection through a confocal stack of the gnathal ganglion (GNG) in frontal view shows bilateral dye injection into the maxillary nerves (MxN, red) and the ascending maxillary medioventral tract (MaxMVT) on the right. Neuropil regions are visualized by phalloidin tagging of actin (green). The dashed line shows the location of the transverse section shown in (**b)** and (**c)**. (**b**) Transverse view of the GNG from (**a**), with the MaxMVT visible in red. The dashed box outlines the higher‐resolution region shown in (**c**). (**c**) High‐resolution Airyscan image of the MaxMVT reveals individual axons. The dashed white line approximates the MaxMVT boundary. (**d**) Projection through a confocal stack of the cerebral ganglion (CRG) in frontal view following bilateral dye injection into the maxillary nerves (orange). Tracts containing dye ascend through the circumesophageal connective (CEC) from the GNG to the GLs. The dashed line shows the location of the transverse section shown in (**e)** and (**f)**. (**e**) Transverse view of the circumesophageal connectives shown in (**d)**, with the dashed box outlining the region shown in (**f)**. (**f**) High‐resolution Airyscan image of CEC, with individual axons visible. The dashed line approximates the boundary of the ascending tract. (**g**) Violin plot of measured axon diameters from three MVTs, with the median shown by a dashed line. (**h**) Projection through a confocal stack of the GNG showing profiles of both pairs of maxillary and labial MVTs (LabMVT). The dashed line shows the location of the section shown in (**i)**. (**i**) Transverse view of the GNG showing cross sections of the maxillary and labial MVTs. (**j**) Violin plot of measured MVTs from four different gnathal ganglia, with the mean shown by a dashed line. Scale bars: 200 µm (**a**, **b**, **d**, **e)**, 100 µm (**h)**, and 5 µm (**c**, **f)**.

We had observed that labeling in the maxillary and labial nerves was more extensive than in the medial ventral tracts, indicating that only a small portion of palp neurons projects from the GNG to the GL. To examine the pathways from sensory centers in the GNG to the GL, we analyzed transverse sections of the medial ventral tracts in ganglia with stained maxillary palp nerves, focusing on axons projecting from the palps to the GL (Figure 5). Using high‐resolution Airyscan confocal microscopy, we counted individual labeled axons in three maxillary medial ventral tracts—one located within the GNG itself (Figure 5a–c) and two in the circumesophageal connectives between the GNG and the cerebral ganglia (CRG) (Figure 5d–f). Across these three tracts, we counted 263 labeled axons. The median axon diameter of this slightly skewed distribution was 0.47 µm (Figure 5g). This value was comparable to that reported previously at the terminal end of the maxillary nerve in the locust *S. gregaria*, where ∼90% of axons had diameters of 0.5 µm or less, indicating that afferent axon diameters increase only slightly as they travel from the sensory periphery through the CNS (Blaney and Chapman 1969b). We also examined the size of the medial ventral tracts themselves (Figure 5h–j). In transverse sections of 15 tracts in four gnathal ganglia, the maxillary and labial tracts had similar areas (147.7 ± 7.3 µm^2^, population mean ± SE; *p* = 0.955, Wilcoxon rank sum test for differences between maxillary and labial) that were much smaller than the fused maxillary palp nerve measured at the tip of the palp in *S. gregaria* (cross‐sectional area of about 1000 µm^2^ [Blaney and Chapman 1969a]). These results suggest that most axons originating in the palps do not extend into the medial ventral tracts.

To estimate the number of axons extending into these tracts, we took two approaches. First, an upper bound measure based on density assumptions (see Section 2) yielded an estimate of about 334 axons per medial ventral tract. This approach likely overestimates the true number, as the median axon diameter measured in this tract fell within the top decile of those measured at the palp tips (Blaney and Chapman 1969b). This suggests that axons increase slightly in diameter from the palp to the GNG, meaning fewer would occupy the same cross‐sectional area. Second, by directly counting the axon profiles in our most complete dye fill of the medial ventral tract, we identified a total of 127 (Figure 5c). This number is likely an underestimate, as some axons may not have been filled completely. These estimates likely bracket the true number of axons projecting from each palp to the GL, suggesting it is innervated by only 5%–15% of the approximately 2300 neurons in the palp tip.

Together, these anatomical findings show the GL receives input from only a small subset of chemosensory neurons on the palp, providing additional evidence that the GL serves a specialized sensory function.

### 3.5 Axons From Basiconic Sensilla on the Palps Innervate the GL

**FIGURE 6:**
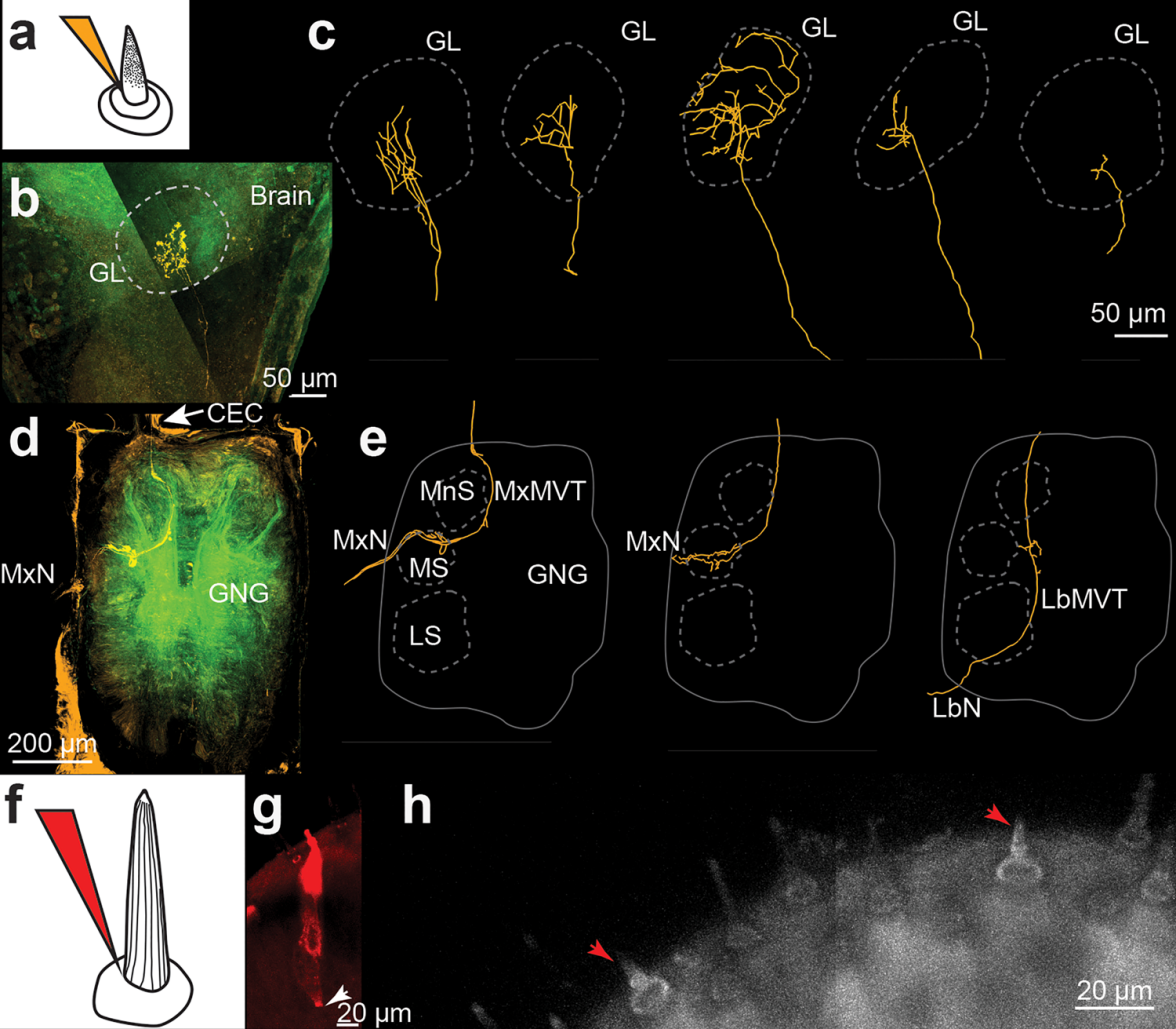
Dye injections into individual sensilla reveal that only basiconic olfactory neurons project to the glomerular lobe. (a) Filling of a single basiconic sensillum with neurobiotin. (b) A projection through a confocal stack of the locust brain in frontal view reveals the innervation targets of olfactory sensory neurons from a single basiconic sensillum, including axon terminals in the microglomeruli of the GL. Neuropil regions are visualized by phalloidin tagging of actin (green). (c) Reconstruction of axonal projections from single basiconic fills from the maxillary palp in the GL, as well as of a single neuron reconstructed from a mass fill of the labial palp (third from the left). Note that the leftmost fill labeled three axons from the single sensillum. (d) A projection through a confocal stack of the gnathal ganglion (GNG) of the same fill as shown in (b). Axons of individual neurons are visible entering the circumesophageal connective (CEC). (e) Reconstruction of the GNG portions of the first three neurons in (c). A few collaterals are visible innervating the maxillary sensory center (MS), though none are visible in the labial sensory center (LS) or mandibular sensory center (MnS), even for axons originating in the labial nerve. (f) Filling of a single chaetic sensillum with neurobiotin. (g) A projection through a confocal stack of the locust palp reveals that neurobiotin did not penetrate into the afferent axons (white arrowhead) of neurons associated with chaetic sensilla. (h) A projection through a confocal stack of the locust palp shows that only basiconic sensilla (red arrowheads) were labeled by the dye fill of the ipsilateral circumesophageal connective. Scale bars: 200 µm (d, e), 50 µm (b, c), and 20 µm (g, h).

We had established that only a small portion of chemosensory neurons on the palps sent processes to the GL. Since mass staining from the palp tip labeled all neurons in both the maxillary and labial nerves, we could not employ this method to distinguish whether these nerves transmit gustatory or olfactory signals to the GL. To investigate whether the GL receives input from basiconic sensilla on the maxillary palps, we injected dye into basiconic sensilla on the maxillary palps. In four of 73 experiments, axonal processes were filled sufficiently to trace their projections. These neurons branched sparsely within the GNG, typically extending a few collateral processes to the MS. But, in all cases, these neurons sent axons along the maxillary medial ventral tracts, exited the GNG via the circumesophageal connectives, and innervated the ipsilateral GL (Figure 6a,b,d). These results show the GL is innervated by neurons housed in basiconic sensilla.

Additional evidence supporting this conclusion came from nerve fills and retrograde labeling. Some labial nerve fills labeled individual ascending neurons (i.e., left side of Figure 3b) whose morphologies resembled those of neurons from basiconic sensilla within the maxillary nerve, exhibited very little innervation of the LS, and extended only a few collaterals as their axons passed through the MS (third from left in Figure 6c,e). Attempts to label neurons in chaetic sensilla yielded partial fills that extended only to the somata, 50–80 µm beneath the sensilla (Figure 6f,g). However, in retrograde fills from the circumesophageal nerve, we were able to identify 10 labeled sensilla on the ipsilateral palp of three preparations (Figure 6h). All 10 labeled sensilla were basiconic, even though basiconic sensilla only comprise a small fraction of the chemosensory hairs on the tip of the palp. This result, combined with our earlier observation that tracer uptake is not selective for specific types of axons in the palp nerve, strongly supports the hypothesis that afferent neurons from chaetic sensilla do not ascend to the GL and that, instead, the processes projecting from the palps to the GL all originate in basiconic sensilla.

We next examined how individual afferent neurons innervate the GL. In total, four successful basiconic sensillum fills labeled six olfactory neurons, with an additional neuron labeled by mass fill of the labial nerve. We reconstructed these neurons (Figure 6c,e) and counted the number of microglomeruli innervated by each. Previous work based upon Golgi stains showed neurons innervated, on average, only one or two microglomeruli within the GL (Ignell et al. 2000). However, most of our reconstructions revealed more extensive multi‐glomerular innervation, with afferent neurons innervating on average 10.9 ± 4.9 microglomeruli (mean ± SE, median = 7). Thus, while some afferents from the palps innervate only one or two glomeruli, most innervate many more. In the antennal system, by comparison, each afferent neuron from basiconic sensilla typically contacts two to six microglomeruli (Hansson et al. 1996; Ignell et al. 2001; Jiang et al. 2024).

Finally, we compared the number of axons projecting into the GL with the number of olfactory neurons estimated to reside in palp basiconic sensilla. We found that, although not all palp axons project to the GL, all neurons traced from the palp's basiconic sensilla do so, consistent with previous observations in *L. migratoria* (L. Sun et al. 2022). Further, the number of axons traveling from each palp to the GL (5%–15% of 2300 axons, or 115–345 total axons) roughly matches the number of neurons in basiconic sensilla per palp (∼165 neurons per palp, or ∼330 neurons per GL).

Together, these findings show that the GL is primarily, and likely exclusively, innervated by axons from basiconic sensilla. Because basiconic sensilla house OSNs, our anatomical findings suggest the GL is specialized for olfactory, rather than gustatory, processing.

### 3.6 Second‐Order Neurons Project From the GL to the Mushroom Body and the Lateral Protocerebrum

**FIGURE 7:**
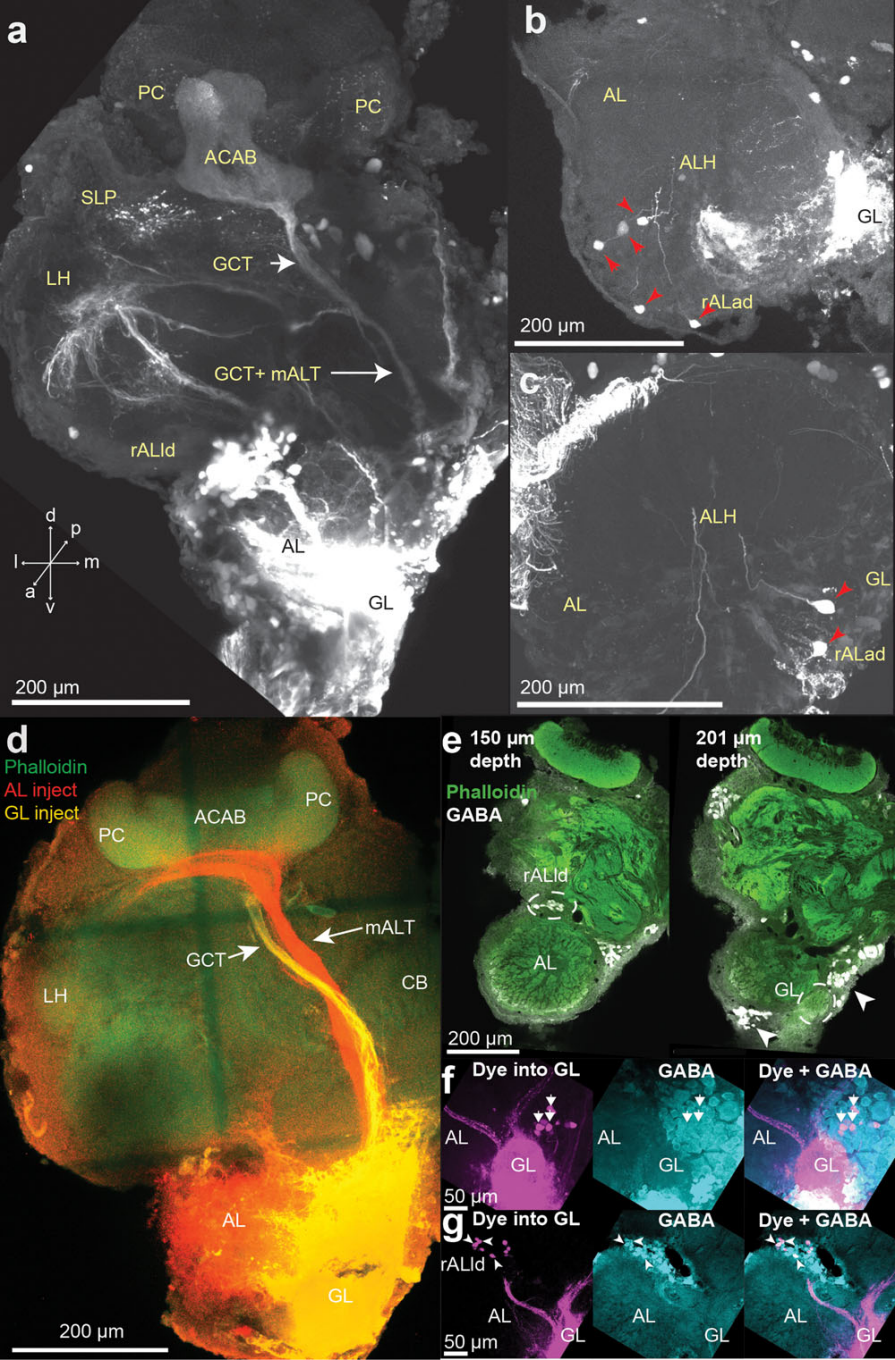
Dye injections reveal connectivity of the glomerular lobe to other parts of the locust brain. (a) A projection through a confocal stack of the locust brain in frontal view shows tracts filled by unilateral dye injection into the GL. Major neuropil regions, tracts, and clusters of cell bodies labeled by the dye injection include the accessory calyx bulb (ACAB), the superior lateral protocerebrum (SLP), the glomerular lobe‐calycal tract (GCT), the medial antennal lobe tract (mALT), the AL, the GL, and the soma cluster in the cell body rind laterodorsal to the antennal lobe (rALld). (b, c) Maximum projection images through the ALs of two different brains labeled by dye injection into the GL. Red arrows indicate the locations of somata in the anterodorsal antennal lobe rind cluster (rALad), with primary neurites extending unbranched through the antennal lobe hub (ALH) and into the GL. (d) Dye injection into the AL (red) shows the mALT, which connects projection neurons of the antennal olfactory system in the AL to higher‐order olfactory centers in the mushroom body primary calyx (PC) and lateral horn (LH). Injection into the GL (orange) shows the distinct GCT associated with palp sensory neurons, connecting the GL with the accessory calyx bulb (ACAB) in the mushroom body. Note that both tracts run together before splitting at the level of the central body (CB). Green indicates neuropil stained with phalloidin. (e) Confocal images of a locust brain stained against gamma‐aminobutyric acid (GABA, white), at depths of 150 and 201 µm into the tissue. Phalloidin (green) allows visualization of neuropil. (f) Colocalization of dextran injected into the GL with anti‐GABA staining: (left) dextran dye injection (magenta) into the GL stains nearby somata (arrows); (middle) anti‐GABA staining (cyan), showing that a large number of somata surrounding the GL are GABA+; (right) color merge showing that the nearby somata innervating the GL are GABA+. (g) Dextran dye injection into the GL stains rALld somata (magenta, left); (middle) GABA+ immunolabeling of somata in the same cluster (cyan); (right) color merge showing that some of the dextran‐labeled somata were also GABA+ (white). Scale bars: 200 µm (a–e) and 50 µm (f, g).

After establishing that the GL receives olfactory input, we next characterized its downstream projection pathways. Dextran‐conjugated dye injections into the GL (87 injections in 69 animals; Figure 7) revealed a cluster of somata in the rind, located laterodorsal to the AL, known as the rALld (Ito et al. 2014). These neurons project neurites into the GL via a tract that passes through the posterior AL. The stains also revealed axonal processes extending from the GL, forming the GCT (previously known as the tritocerebral tract [Homberg et al. 2004; von Hadeln et al. 2018]). This tract joins the mALT for a short stretch, bringing outputs from glomerular and ALs into close proximity (Figure 7d). Extending dorsally, the GCT separates from the mALT just posterior to the CB, branches laterally, and extends dorsally alongside the PED before broadly innervating the ACAB.

While the great majority of processes labeled by dextran injection into the GL followed this ascending pathway, a few others extended elsewhere, including two to 15 somata in the cell body rind anterodorsal to the AL (rALad) that did not send any processes into AL glomeruli, but instead traveled without branching through the AL hub before innervating the GL (Figure 7b,c). These somata were intermixed with those of the local and projection neurons of the AL. Along the GCT, we also found five to 10 processes that branched out laterally, innervating the SLP (Figure 7a).

To understand how the GL processes information, it is necessary to identify neurotransmitters used by its neurons. The GL is immunoreactive for choline acetyltransferase, likely reflecting the presence of cholinergic input from the chemosensory afferent neurons on the palps (Lutz and Tyrer 1987). Because GABAergic signaling substantially shapes neuronal responses in the olfactory pathway (Stopfer et al. 1997; Laurent 2002; Ray et al. 2020), we stained 48 locust brains with antibodies against GABA (Figure 7e–g). We found GABA‐positive processes throughout the GL, and two clusters of GABA‐positive somata surrounding it (Figure 7e,f). The locations of these two clusters match prior descriptions of local neurons innervating the GL (Satija 1958). We also determined that several neurons adjacent to the GL (in the rALld) were GABA positive (Figure 7e), including some that were also labeled by dye injection into the GL (Figure 7g). Notably, we did not observe GABA staining in either of the GL downstream regions, the ACAB, or the SLP (Figure 7e). These results provide anatomical indications that GABAergic inhibition contributes to processing olfactory information in the GL.

Taken together, these anatomical results reveal a pathway between the GL and higher‐order areas in the cerebrum. Notably, the tracings reveal a projection pathway suited for relaying olfactory information from the palps, via the GL, to the mushroom body and lateral protocerebrum. Further, immunostainings identify key neurotransmitters likely involved in processing these signals.

### 3.7 GL Projection Neurons Comprise at Least Eight Distinct Morphological Classes

**FIGURE 8:**
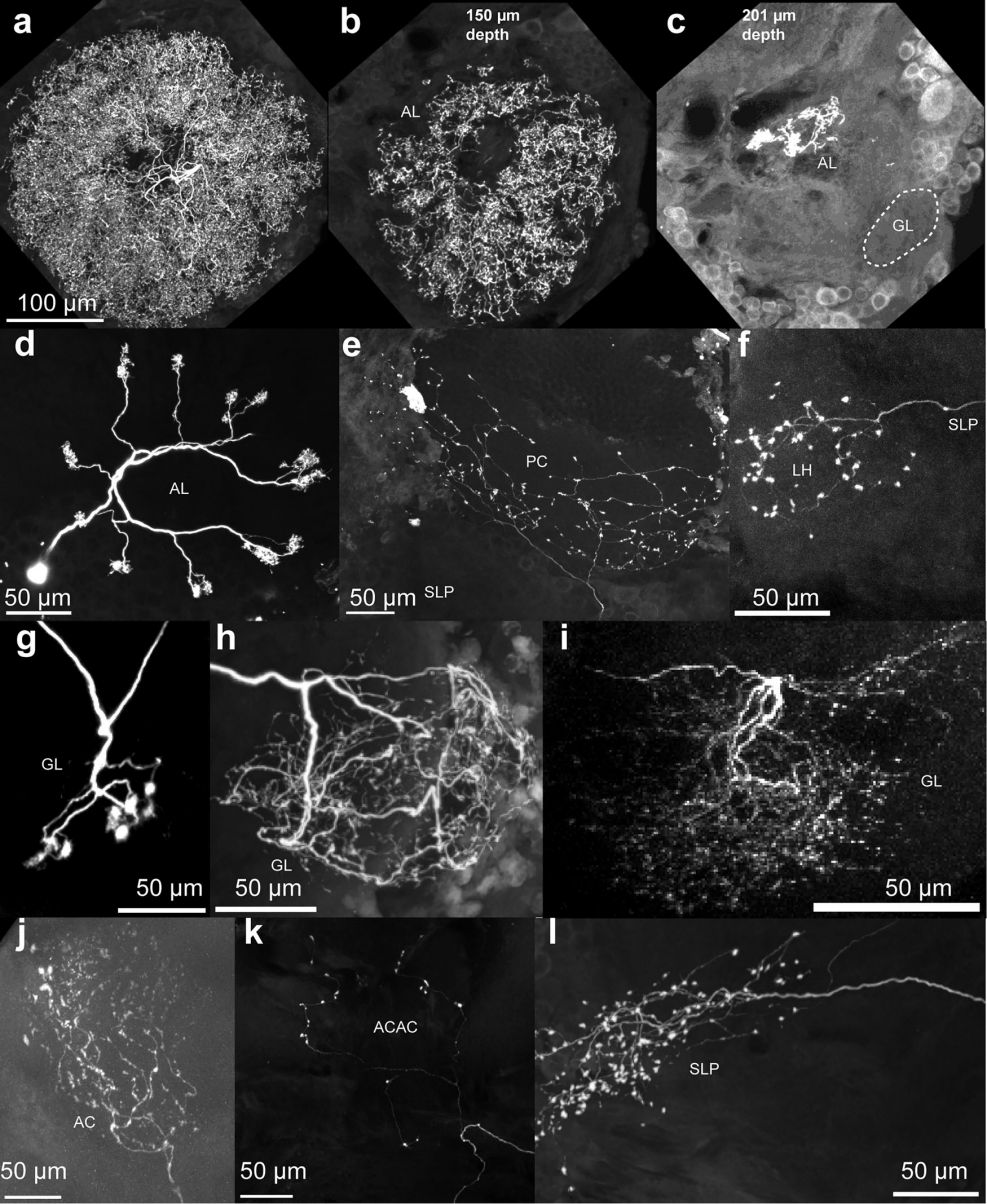
Second‐order palp and antennal olfactory neurons show common innervation patterns. (a) Maximum intensity projection showing the morphology of an AL local neuron (ALLN). There is no discernible differentiation between axonal and dendritic processes, with neither dendritic tufts corresponding to microglomeruli nor varicosities visible. (b) Single slice from a confocal stack of an ALLN, showing that it innervates all regions within the AL. (c) Single slice from a confocal stack at a depth of 201 µm showing that the ALLN innervates the AL, but not the GL. (d) Maximum intensity projection of an antennal lobe projection neuron (ALPN) in the AL, showing the dendrites of the ALPN densely innervating several microglomeruli. (e, f) Maximum intensity projections of the primary calyx and lateral horn, respectively. Synaptic varicosities are clearly visible as swellings within the axonal processes. (g) Maximum intensity projection of class 1 glomerular lobe projection neuron (GLPN) in the GL, showing dendritic branching similar to the ALPN. Note the dense dendritic arbors, matching the size of microglomeruli in the GL. (h, i) Maximum projection of class 3 and class 6 GLPNs, respectively, showing extensive dendritic branching patterns that fill out nearly the entire GL, without obvious innervation of individual microglomeruli. (j) Maximum intensity projection through the accessory calyx bulb of the mushroom body showing the dense axonal arborization of a class 1 GLPN (same neuron as in panel g). Varicosities along the axonal branches are visible. (k, l) Maximum intensity projection through the accessory calyx core (ACAC, k) of the mushroom body, and the superior lateral protocerebrum (SLP, l), showing the axonal arborization and varicosities of a class 3 GLPN (same neuron as in panel h). Scale bars: 100 µm (a–c) and 50 µm (d–l).

We found that GLPNs comprise a diverse population receiving far less convergent input from receptor neurons than projection neurons in the AL. To better characterize the palp olfactory pathway, we injected dye through patch clamp electrodes into 328 somata adjacent to the AL and GL in 179 animals. Although the vast majority of the labeled cells were projection (ALPNs) and local neurons (ALLNs) innervating only the AL (Figures 8a–f and 10a), 21 neurons sent their dendritic processes exclusively into the GLs. We reconstructed these 21 GL neurons and manually grouped them into eight classes based on their morphologies and the regions innervated by their axonal processes (Figure 10b,c).

The dendritic innervation patterns of these GLPNs varied widely across classes. Morphologically, the GL innervation of class 1 GLPNs closely resembled the branching patterns of ALPNs (compare Figure 8d with Figure 8g). Of six reconstructed class 1 GLPNs, each innervated 9.7 ± 1.0 microglomeruli (mean ± SEM). In contrast, the dendritic fields of other classes of GLPNs were more diffuse and spread throughout the entire GL (Figure 8h,i). For comparison, we reconstructed six ALPNs, where we found 14.3 ± 1.9 (mean ± SEM) microglomeruli innervated per neuron, consistent with previous measurements in locusts (Anton et al. 2002; K. Sun et al. 2024; Jiang et al. 2024).

Downstream regions innervated by these neurons include the ACAB (class 1, Figure 8j), ACAR (classes 2 and 5), ACAC (class 3, Figure 8k), the primary calyx (class 2), the SLP (classes 3, 4, 6, and 8, Figure 8l), and the ventral lateral protocerebrum (classes 5–7). Notably, although ALPNs and GLPNs both carry olfactory information, GLPNs, other than class 2, targeted downstream regions separate from those targeted by ALPNs (Figure 10c).

**FIGURE 9:**
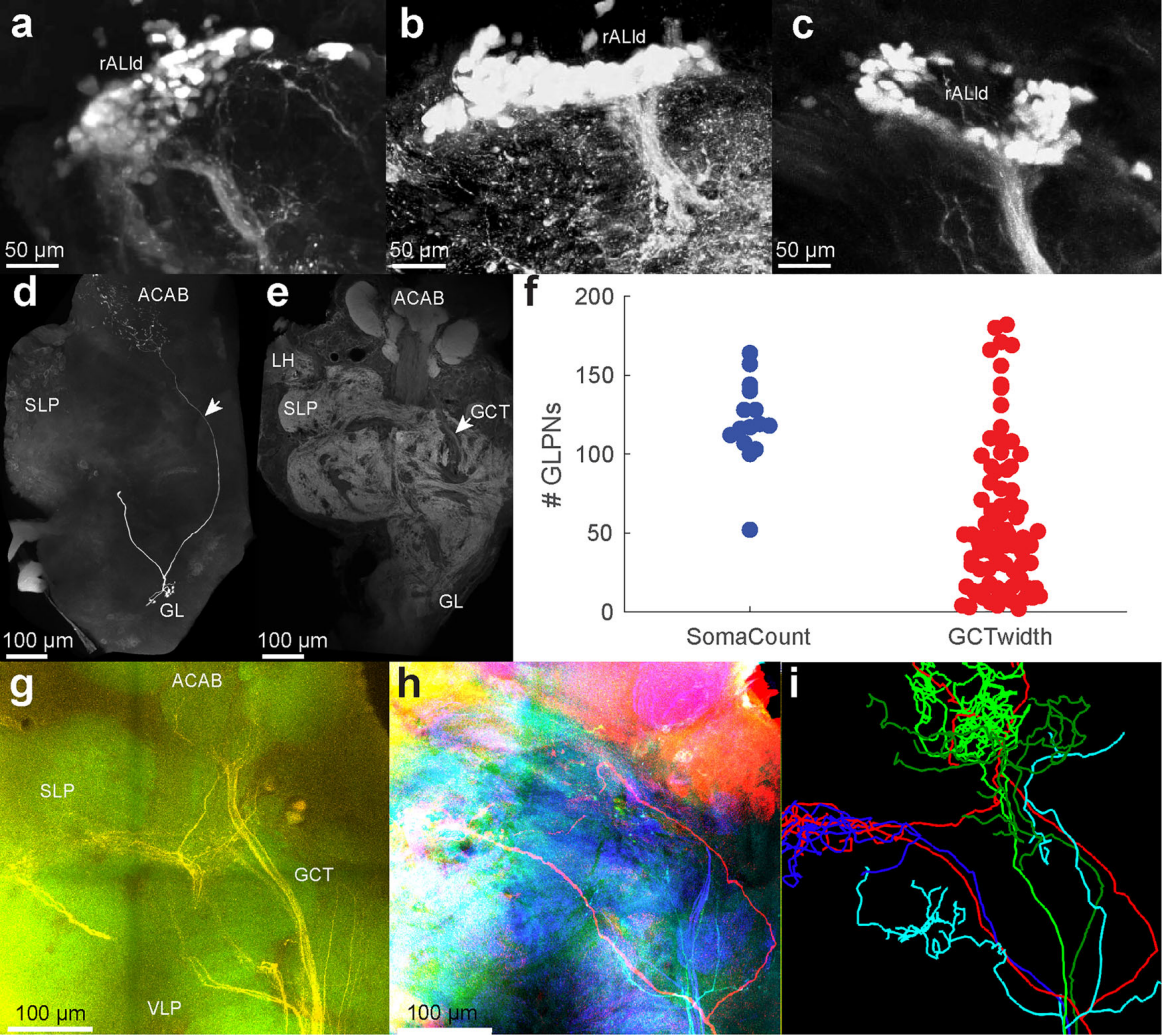
Numbers of glomerular lobe projection neurons. (a–c) Three example maximum intensity projections of the soma in the laterodorsal antennal lobe rind (rALld) as revealed by dextran injection into the GL. (d) Maximum intensity projection from a class 1 GLPN stain. The diameter of the axon was measured at a point where the glomerular lobe‐calycal tract (GCT) separates from the medial antennal lobe tract (mALT) (arrowhead). (e) Maximum intensity projection from the standard brain showing the region where the GCT separates from the mALT (arrowhead). (f) Two methods for estimating the number of GLPNs, from a direct count of labeled rALld soma in dextran injections (blue markers) and circle‐packing calculations comparing reconstructed GLPN axon diameters with GCT diameters (red markers). (g) Maximum intensity projection from a confocal stack of a mass dye fill into the GL. The projection shows the dorsal portion of the protocerebrum and mushroom body, revealing tracts of many GLPNs innervating the mushroom body, ventral protocerebrum, and superior lateral protocerebrum. (h) Overlay of neurobiotin stains of single examples of GLPN classes 1 (light green), 2 (dark green), 3 (red), 4 (blue), and 5 (cyan), all from different animals, aligned to the standard brain. The same region of the dorsal protocerebrum is shown as in (g). (i) Overlay of the reconstructions of the same five neurons as shown in (g). Note that all of the axons outside of the GCT are accounted for by one to three neurons of each of the class 2–5 GLPNs. Scale bars: 100 µm (d, e, g, h) and 50 µm (a–c).

We next sought to determine whether these eight classes comprehensively describe the entire population. Most GLPN classes appeared to consist of relatively few neurons. Five of the eight classes we observed had somata exclusively in the rALad cluster (classes 2–6), and one (class 1) had somata in both the rALad and rALld clusters. Mass fills of the GL revealed no more than 15 somata in the rALad cluster, suggesting that GLPN classes 2–6 likely comprise only a few neurons per hemisphere. A comparison of mass fills with reconstructions of class 1–5 GLPNs (Figure 9g–i) showed that the labeled fibers from the GL that innervate the mushroom body or lateral protocerebrum are accounted for by these identified classes. This indicates that our eight classes are likely to represent all the GL projection neurons.

**FIGURE 10:**
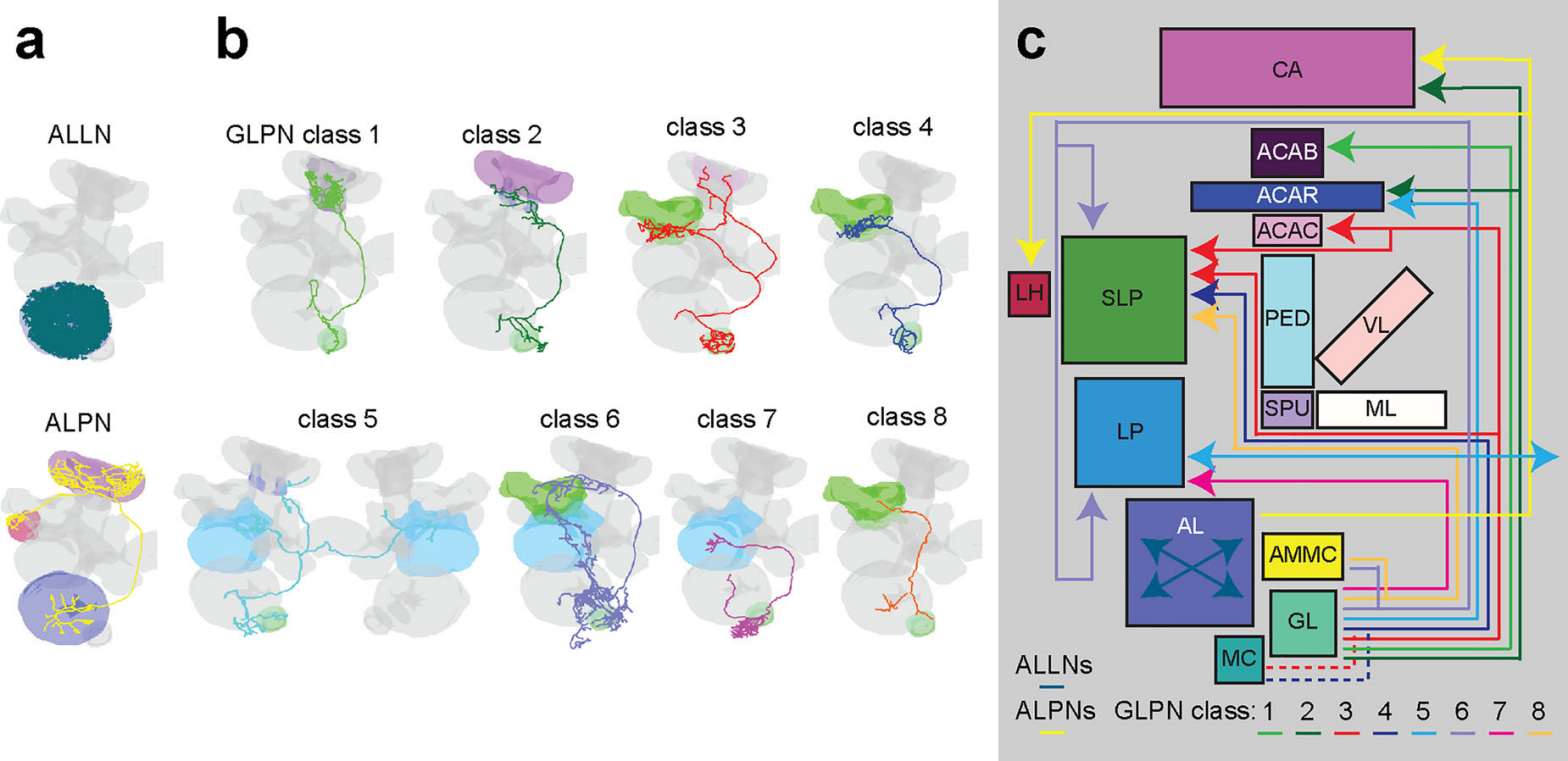
Eight classes of glomerular lobe projection neurons connect the GL to distinct regions of the protocerebrum. (a) Reconstructions of filled AL local neurons (ALLN, upper) and AL projection neurons (ALPN, lower). These are the two main cell types of the AL in the antennal olfactory system, each of which is thought to be represented by only a single anatomical class. The two main brain regions receiving ALPN output, the primary calyx (purple) and the lateral horn (red), are highlighted in color. **(b)** Reconstructed examples of each class of glomerular lobe projection neuron (GLPN). For each neuron class, the GL (green) and the putative postsynaptic target regions of neuropil in the brain are highlighted in color. **(c)** Schematic showing ipsilateral neuropil regions innervated by GLPNs, ALPNs, and ALLNs. Colored arrows represent axons extending from each neuron class to the indicated brain region.

We used two approaches to estimate the total number of class 1 GLPNs, which make up the majority of neurons innervating the GL. We obtained a lower bound estimate of 160 by counting the number of somata in the rALld cluster labeled by dextran injections into the GL (Figure 9a–c). In our set of reconstructed GLPNs, all neurons with soma in the rALld cluster belonged to class 1 (Figure 9f, blue). Next, we obtained an upper bound estimate of 180 by calculating the number of GLPN axons that could fit within the GCT (Figures 9d–f and 10b,c; see Section 2). Our estimate of 160–180 class 1 GLPNs refines previous counts for this population (Ernst et al. 1977; Homberg et al. 2004) and suggests that class 1 cells constitute the vast majority of the GLPN population, leaving at most a few dozen neurons in the remaining classes.

We next examined how sensory input is integrated within the GL by characterizing the overall pattern of neural convergence in the palp pathway and comparing it to that of the antennal olfactory pathway. The GL receives input from about 330 olfactory neurons (Table 1), each of which contacts ∼7 of the lobe's ∼690 microglomeruli (Figure 6b). Thus, on average, each microglomerulus in the GL receives input from ∼3.3 OSNs. On the output side, each of the 160–180 class 1 GLPNs (Figure 9f) contacts ∼10 microglomeruli (Figure 8g), so each microglomerulus sends output to ∼2.5 class 1 GLPNs. We could not determine the exact number of microglomerular contacts for other GLPN classes because their dendritic tufts were not clearly resolved in the GL. Nevertheless, the high density of these tufts suggests that the other GLPNs each contact multiple microglomeruli (Figure 8h,i).

By comparison, the AL receives input from ∼85,000 OSNs (Greenwood and Chapman 1984; Ochieng et al. 1998; Chapman 2002). Because each neuron targets 2–6 microglomeruli (Hansson et al. 1996; Ignell et al. 2001; Jiang et al. 2024) out of a total population of ∼800 (Ernst et al. 1977; Laurent and Naraghi 1994; Ignell et al. 2001; Anton et al. 2002; Ott and Rogers 2010; Jiang et al. 2024), each microglomerulus receives input from ∼200–600 OSNs. Additionally, the AL contains 830–900 ALPNs (Ernst et al. 1977; Leitch and Laurent 1996; Anton et al. 2002), each of which arborizes within ∼15 microglomeruli (Anton et al. 2002; K. Sun et al. 2024; Jiang et al. 2024); consequently, each microglomerulus provides output to ∼16 ALPNs.

These comparisons reveal a fundamental difference in how the two olfactory systems are structured. The antennal system exhibits strong convergence, with many more sensory neurons at its periphery pooling onto a much smaller population of projection neurons, leading to a convergence ratio at the microglomerular level of between 10:1 and 40:1. In contrast, the palp system shows minimal convergence: the number of projection neurons in the GL is similar to the number of afferent neurons reaching it, with a convergence ratio at the GL microglomerular level of ∼4:3.

Together, our findings demonstrate that olfactory processing in the GL involves relatively little pooling of sensory input. Instead, information is transmitted through multiple distinct classes of projection neurons to separate regions downstream. Except for class 2 GLPNs, which target the primary calyx, the output pathways of the GLs remain anatomically separate from those of the antennal system, raising the possibility that olfactory information originating in the palps and the antenna serves different functions.

### 3.8 Third‐Order Neurons Project From the Accessory Calyx to the Output Lobes of the Mushroom Body

**FIGURE 11:**
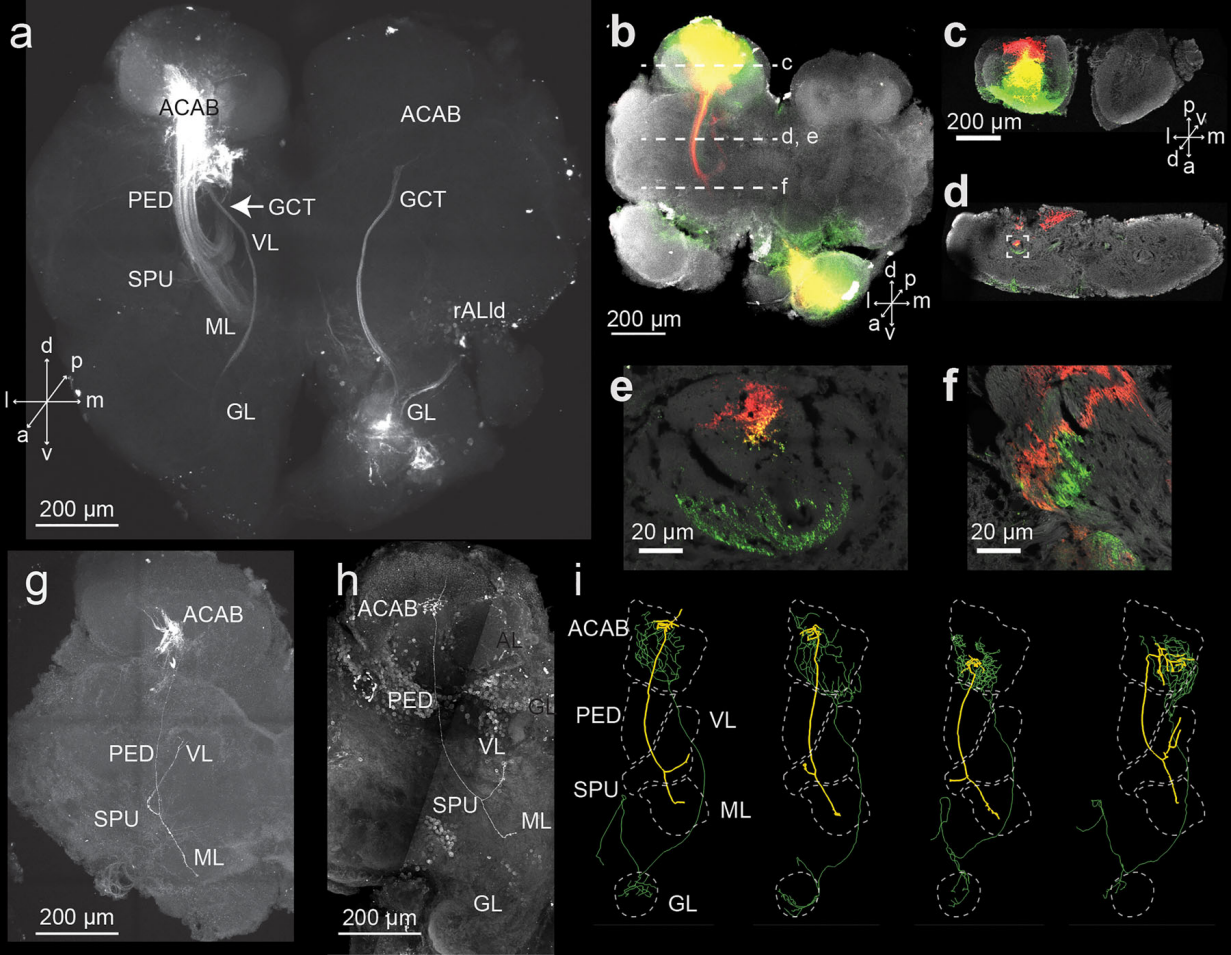
Dye injections into the mushroom body reveal downstream targets of GLPNs. (a) Projection through a confocal stack showing tracts filled by dye injections into the accessory calyx bulb (ACAB, left side) and GL (right side) of the same locust brain. The prominent glomerular lobe‐cerebral tract (GCT), connecting the GL with the ACAB of the mushroom body, is clearly visible on both sides of the brain. On the left, many axons are visible descending from the ACAB through the mushroom body pedunculus (PED) into the medial lobe (ML), vertical lobe (VL), and spur (SPU). (b) Maximum intensity projection from a confocal stack of dual‐labeled mass dye fills, with dextran‐conjugated Alexa Fluor 633 dye (red) loaded into regions innervated by the palp olfactory system: the mushroom body accessory calyx (left) and GL (right), and dextran‐conjugated Alexa Fluor 568 dye (green) loaded into the mushroom body primary calyx (left) and antennal lobe (right). Dashed lines indicate the levels of horizontal sections shown in panels c–f. (c) Horizontal section through the locust mushroom body showing accessory calyx neurons labeled by dye injection in red and primary calyx neurons labeled by dye injection in green. (d) Horizontal section through the protocerebrum showing compartmentalization within the mushroom body pedunculus, with red dye constrained to the posterior section of the pedunculus, and green dye present in a large crescent‐shaped compartment on the anterior side. The stippled box indicates the region shown in panel e. (e) High‐resolution Airyscan image of the mushroom body pedunculus shown in panel d. (f) High‐resolution Airyscan image of the mushroom body median lobe in horizontal section (a different animal than in panels b–e). Alternating bands are innervated by processes originating in the accessory calyx (red) and primary calyx (green). (g) Projection through a confocal stack of different brains showing less‐dense labeling in the mushroom body following dye injection into the ACAB. Individual KCs innervating the accessory calyx are visible. (h) Projection through a confocal stack showing a single accessory calyx Kenyon cell (ACKC) stained via a patch clamp electrode. (i) Reconstruction of four ACKCs (gold), along with reconstructions of class 1 glomerular lobe projection neurons (green). Note that the middle two reconstructions send branches into the SPU rather than the VL. Scale bars: 200 µm (a–d, g, h) and 20 µm (e, f).

To identify third‐order neurons in the palp olfactory pathway and determine whether their inputs originate primarily in the GL, we injected dye into the GL and the ACAB of opposite hemispheres in the same animal (Figure 11a). Notably, all descending fibers labeled by injections in the accessory calyx were in the GCT, the same pathway labeled by injections into the GL. The selective labeling of the GCT via retrograde staining confirms that the pathway originating from the rALld soma cluster projects to the accessory calyx after passing through the GL. The retrograde tracings further confirm that the innervation of the accessory calyx (Figure 7a) consists exclusively of processes from the GL (rather than from possible off‐target injections near the GL), consistent with previous findings (Weiss 1981).

Dye injections into the ACAB revealed a large population of intrinsic mushroom body neurons that sent dendrites laterally within the accessory calyx and axons downward through the pedunculus into the VL and ML, the mushroom body's main output sites. Because some mass dye fills labeled only a few neurons in the mushroom body (Figure 11g), it was possible to view the morphologies of individual third‐order neurons in the palp olfactory pathway. Although these neurons resemble Kenyon cells (KCs) that receive AL input, the dendritic fields of palp‐associated KCs are located in the accessory calyx rather than the primary calyx.

For a detailed analysis of individual accessory calyx KCs, we targeted somata adjacent to the calyx, dye‐filled them through patch electrodes, and reconstructed their 3D morphologies (Figure 11h). We found the innervation patterns of these neurons overlapped with those of class 1 GLPNs (Figure 11i). Some reconstructed accessory calyx KCs resembled those labeled by mass fills, with large branches innervating both the VL and ML (Figure 11g,h). But other accessory calyx KCs sent branches to the SPU rather than the vertical lobe (middle panels in Figure 11i). To our knowledge, primary calyx KCs receiving antennal input have not been reported to innervate this area, raising the possibility that the spur receives olfactory input exclusively from the palps.

We used dual labeling experiments to further compare the pathways followed by primary and accessory calyx KCs. We found KCs originating in the primary calyx remained in the anterior‐most crescent‐shaped compartment of the pedunculus, while those originating in the accessory calyx were confined to the posterior compartment (Figure 11b–f). This segregation was maintained through the ML, where processes from primary calyx KCs and accessory calyx KCs formed alternating bands (Figure 11f). These results are consistent with and extend prior reports that the locust pedunculus contains separate areas for inputs from the different calyces (Weiss 1981; von Hadeln et al. 2018).

Together, our results establish that the third‐order neurons of the palp olfactory pathway, the accessory calyx KCs, are morphologically analogous to, yet physically segregated from, the primary calyx KCs of the antennal olfactory system. Although both pathways process the same sensory modality and traverse the brain via neighboring tracts, their projections remain strictly separated throughout the deutocerebrum and TC, along the GCT and mALT, and within the mushroom body and the adjacent LH/SLP.

## 4 Discussion

### 4.1 Chemosensory Sensilla Located on the Locust Palp Give Rise to Separate Olfactory and Gustatory Neural Pathways

Our anatomical study of the locust *S. americana* reveals that olfactory sensilla on the maxillary and labial palps initiate a second olfactory system separate from the main olfactory system associated with the antennae. The palp gustatory system follows a distinct pathway within the central nervous system. Our results show that projections from neurons in the relatively few olfactory sensilla on the palps (∼3% of the total population) pass through the GNG largely unbranched before innervating the GL in the cerebral ganglion. Notably, although a pathway from the palps to the GL and subsequently to the mushroom body has long been recognized, it has been classified as gustatory rather than olfactory (Jawlowski 1954; Ernst et al. 1977; Weiss 1981; Ignell et al. 2000; Homberg et al. 2004; Frambach and Schurmann 2004; Farris 2008; von Hadeln et al. 2018), probably because most of the neurons on the palps are gustatory. Previous studies demonstrated that neurons from olfactory sensilla project directly to this region (L. Sun et al. 2022) and that neurons expressing the olfactory co‐receptor ORCO are distributed widely throughout the lobe (Jiang et al. 2024), suggesting the GL participates in olfaction. Our results extend these findings by showing the GL is mostly, if not entirely, olfactory.

Most chemosensory neurons in the palps are gustatory and innervate dedicated neuropils within the GNG. Additional gustatory sensilla on other head structures, including the antennae, labrum, and mandibles, send their neurons to separate neuropils in the GNG. We found that these additional gustatory inputs, like those from the palps, do not converge upon the GL and may be integrated elsewhere, perhaps within the GNG.

Our results challenge the existing view that olfactory and gustatory pathways travel in parallel from the sensory periphery to the mushroom bodies. Rather, our results show that the pathway originating in the antennae and projecting to the deuterocerebrum and the pathway originating in the palps and projecting to the TC are both olfactory. These olfactory pathways run in parallel and target separate regions of the mushroom bodies. Whether these two olfactory paths serve distinct functions remains to be determined.

Our results also challenge the prevailing view that gustatory and olfactory inputs target separate compartments within the orthopteran mushroom body. This perspective, which underpins theories of strict modality‐based segregation in the calyces (Farris and Strausfeld 2003; Frambach and Schurmann 2004; Farris 2008; Strausfeld et al. 2009; von Hadeln et al. 2018), is rooted in the assumption that the accessory calyx, fed by the GCT, is a dedicated gustatory center. However, we establish that the GL receives input exclusively from palp olfactory neurons. While it remains unclear how palp‐derived gustatory information is integrated into the mushroom body via the GNG, associative learning studies in locusts provide a plausible alternative, that the gustatory information is conveyed to the mushroom body as a reward signal (Cassenaer and Laurent 2012). Such an arrangement would require the mushroom body to receive only reinforcement signals from, for example, the well‐characterized octopaminergic dorsal unpaired median (DUM) neurons of the GNG (Braunig 1991), rather than detailed information about tastes. Consequently, separate compartments in the mushroom body may not be necessary to support multimodal behaviors like associative learning.

### 4.2 Antennal and Palp Olfactory Pathways Are Constructed From Similar Structures

Our comparative analysis of the structures of palp and antennal olfactory systems in locusts revealed that both are constructed from nearly identical components.

The initial stages of olfactory anatomy in the antennal and palp circuits are similar. In both systems, multiporous olfactory sensilla each house several OSNs (Figure 1d,e,k). In all, each antenna has about 100,000 ORNs (Chapman 1982), distributed within ∼4000 coeloconic, trichodic, and basiconic sensilla (Chapman 1982; Greenwood and Chapman 1984; Ochieng et al. 1998; Ochieng and Hansson 1999; Chapman 2002). By contrast, the palps contain only about 330 OSNs per side, distributed into ∼11 basiconic sensilla on each of the maxillary and labial palps (Table 1) (Blaney 1977; Jin et al. 2006; L. Sun et al. 2022). Notably, the set of OR proteins expressed in palp OSNs is, with a single exception, a subset of those found in the antennae (Xu et al. 2013; Z. Wang et al. 2015; H. Li, Wang, et al. 2018; Lemke et al. 2020; Boronat‐Garcia et al. 2022).

The two pathways also feature analogous primary neuropils, the AL and the GL. In the locust, both the AL (Ernst et al. 1977; Laurent and Naraghi 1994; Anton and Hansson 1996; Ignell et al. 2001) and the GL (Jawlowski 1954; Ernst et al. 1977; Ignell et al. 2000; von Hadeln et al. 2018) are composed of microglomeruli. Afferent fibers entering the respective neuropils each innervate multiple microglomeruli (Figure 6b,c) (Hansson et al. 1996; Ignell et al. 2000; Ignell et al. 2001; L. Sun et al. 2022), and PNs in both pathways send dendritic arbors into multiple glomeruli (Figure 8d,g) (Ernst et al. 1977; Laurent and Naraghi 1994; Anton and Hansson 1996; Ignell et al. 2000; Ignell et al. 2001). In the antennal olfactory system, GABAergic local neurons innervate the entire AL and transmit information and timing signals across its glomeruli (MacLeod and Laurent 1996; Leitch and Laurent 1996; Laurent 2002; Raman et al. 2010) (Figure 8a–c). Less is known about the local neurons associated with the GL. But, neurons with somata adjacent to and extending processes into it have been reported (Satija 1958; Ernst et al. 1977), and our results show some of these neurons stain positively for GABA. Some of these neurons may also project to other brain regions (Figure 7e–g).

Although the components of the two olfactory systems are similar, we found that patterns of neural convergence within the AL and the GL are dramatically different. Where the antennal system has tens of thousands of afferent neurons converging onto hundreds of projection neurons, the palp system has ∼330 afferents converging onto ∼160–180 projection neurons. The different convergence patterns are also apparent at the microglomerular level, where the antennal system has a convergence ratio of afference onto microglomeruli of ∼10–40:1 and the palp system has a convergence ratio of ∼4:3. It will be interesting to determine how this dramatic difference in convergence patterns affects the functions of the two olfactory systems.

### 4.3 Locust Palp and Antennal Olfactory Circuits Are Anatomically Segregated Through Multiple Layers of Processing

Although the locust antennal and palp olfactory systems mediate the same sensory modality initiated by the same OR proteins, our experiments, including single‐neuron dye fills, show the two pathways are completely separate through the first three layers of processing (Figures 3, 4a, 7d, 8, 10, and 11). Our results revise earlier work in orthopterans, which characterized these pathways as olfactory and gustatory, respectively, based on histological treatments and mass staining (Jawlowski 1954; Satija 1958; Ernst et al. 1977; Weiss 1981; Homberg et al. 2004; Frambach and Schurmann 2004; Farris 2008).

Yet, behavioral evidence suggests the two olfactory systems do eventually converge. The palp opening response (POR) assay (Simões et al. 2011) shows locusts can detect an odorant through their antennae when the palp tips are covered in paint (Saha et al. 2015; Nizampatnam et al. 2022; Chandak and Raman 2023), or through the palps after both antennae are removed (Zhang et al. 2017). The fact that stimulating either olfactory organ elicits the same behavioral response suggests that the two olfactory pathways interact at the level of the motoneurons controlling palp movement, if not earlier.

### 4.4 The Olfactory Circuitry of the Palps in Locusts Differs Markedly From That of Flies and Other Holometabolous Insects

The anatomical segregation of the palpal and antennal olfactory pathways in the locust contrasts sharply with the organization of the analogous pathways in *Drosophila*. *Drosophila melanogaster* and most non‐Coleopteran holometabolous insects have no GL (Anton and Homberg 1999; Farris 2008; Dippel et al. 2016), and OSNs in palp sensilla project directly to distinct glomeruli within the medioventral posterior AL (Kent et al. 1986; Couto et al. 2005). Within the fly AL, olfactory information is shared between antennal and palp glomeruli by excitatory LNs and other neurons (Yaksi and Wilson 2010; Shimizu and Stopfer 2017), and the GABA‐mediated inhibition that drives oscillations in PNs is coordinated via LNs that innervate both palp and antennal glomeruli (Tanaka et al. 2009; Chou et al. 2010). Thus, in flies, palp and antennal olfactory pathways are functionally integrated so that PNs and LNs receive information from both olfactory organs and participate in AL‐wide odor‐elicited oscillations. In addition, in flies, AL glomeruli receiving inputs from the palps or antennae send converging projections to the same groups of KCs in the mushroom body (F. Li et al. 2020). Thus, in the fly, olfactory information from the antennae and palps is structurally and functionally integrated into a single olfactory system, beginning in the AL. *Drosophila* evolved more recently than Orthoptera, and it has been suggested that the evolutionary integration of the palp and antennal olfactory systems was driven by the increasing specialization of mouthparts (Nel et al. 2018). Behavioral studies in locusts show that their palps play a role in food selection (Blaney and Chapman 1970; Blaney 1975; Blaney and Duckett 1975, 1981; Blaney and Simmonds 1985; Chapman and Sword 1993). Accordingly, with the evolutionary emergence of piercing, sucking, and lapping mouthparts in more derived insects, the role of the palps in food selection may have been reduced (Bernays and Simpson 1982). In flies, this shift may have freed the palp olfactory system from an exclusive role in feeding, enabling it to participate in the broader processes of odor identification and classification—functions that, in insects like locusts, are commonly attributed to the antennal olfactory system. Electrophysiological experiments can test this hypothesis by determining whether the palp olfactory system of locusts and other insects with nonspecialized mouthparts is specifically responsive to food‐related odors.

## Author Contributions

Z.N.A., B.K., and M.A.S. designed research. Z.N.A., B.K., K.S., E.N., and V.N. performed research. M.A.S. contributed unpublished reagents/analytic tools. Z.N.A., B.K., and E.N. analyzed data. Z.N.A. and M.A.S. wrote the article.

## Funding

This research was supported in part by an intramural grant from the Eunice *Kennedy Shriver* National Institute of Child Health and Human Development, national Institutes of Health (1ZIAHD008760‐20 to M.A.S.). This research was also supported in part by the National Heart, Lung, and Blood Institute (NHLBI) Electron Microscopy Core (ZIC‐HL005906‐18 to Valentina Baena and Zulfeqhar A. Syed).

## Conflicts of Interest

The authors declare no conflicts of interest.

## Data Availability

All data supporting the findings of this study have been deposited in Zenodo and are available at https://doi.org/10.5281/zenodo.20146646. In addition, the *Schistocerca americana* standard brain generated for this study, as well as all individual reconstructed neurons, has been made available via the Insect Brain Database: https://www.insectbraindb.org/app/species/53.
